# A Weakened Immune Response to Synthetic Exo-Peptides Predicts a Potential Biosecurity Risk in the Retrieval of Exo-Microorganisms

**DOI:** 10.3390/microorganisms8071066

**Published:** 2020-07-17

**Authors:** Katja Schaefer, Ivy M. Dambuza, Sergio Dall’Angelo, Raif Yuecel, Marcel Jaspars, Laurent Trembleau, Matteo Zanda, Gordon D. Brown, Mihai G. Netea, Neil A. R. Gow

**Affiliations:** 1 The Aberdeen Fungal Group, School of Medicine, Medical Sciences & Nutrition, Institute of Medical Sciences, University of Aberdeen, Foresterhill, Aberdeen AB25 2ZD, UK; I.M.Dambuza@exeter.ac.uk (I.M.D.); gordon.brown@exeter.ac.uk (G.D.B.); n.gow@exeter.ac.uk (N.A.R.G.); 2 Medical Research Council Centre for Medical Mycology at the University of Exeter, Geoffrey Pope Building, Stocker Road, Exeter EX4 4QD, UK; 3 Institute of Medical Sciences, University of Aberdeen, Aberdeen AB25 2ZD, UK; s.dallangelo@abdn.ac.uk (S.D.); m.zanda@lboro.ac.uk (M.Z.); 4 Iain Fraser Cytometry Centre (IFCC), University of Aberdeen, Foresterhill, Aberdeen AB25 2ZD, UK; R.Yuecel@exeter.ac.uk; 5 Centre for Cytomics, Geoffrey Pope Building, University of Exeter, Stocker Road, Exeter EX4 4QD, UK; 6 Marine Biodiscovery Centre, Department of Chemistry, University of Aberdeen, Meston Walk, Aberdeen AB24 3UE, UK; m.jaspars@abdn.ac.uk (M.J.); l.trembleau@abdn.ac.uk (L.T.); 7 Sir David Davies Building, Centre for Imaging Science, School of Science, Loughborough University, Loughborough LE11 3TU, UK; 8 Department of Internal Medicine, Radboud University Medical Center, 6525 GA Nijmegen, The Netherlands; Mihai.Netea@radboudumc.nl; 9 Department for Genomics & Immunoregulation, Life and Medical Sciences Institute (LIMES), University of Bonn, 53115 Bonn, Germany

**Keywords:** unusual amino acids, exobiology, infection risk, planetary protection, space travel, immune response

## Abstract

**Simple Summary:**

We tested the immune response of T cells of the mammalian immune system towards protein antigens that includes the unusual amino acids isovaline and α-aminoisobutyric. Those amino acids have been found in high abundance on carbonaceous meteorites but are extremely rare in proteomes of earth organisms. We hypothesised that proteins of non-terrestrial alien life forms might contain such amino acids and tested whether chemically synthesised “exopeptides” that contain these amino acids could be detected by the immune system. Our assays, based on the responses of CD8^+^ T cells to these exopeptides, indicated that antigen cleavage, processing, and subsequent T cell activation still occurred, but were less efficient than the response to control peptides that lacked these amino acids. We therefore speculate that the encounter of putative exo-microorganisms of an unusual antigenic repertoire might pose an immunological risk for space missions aiming to retrieve potentially biotic samples from exoplanets and moons.

**Abstract:**

The discovery of liquid water at several locations in the solar system raises the possibility that microbial life may have evolved outside Earth and as such could be accidently introduced into the Earth’s ecosystem. Unusual sugars or amino acids, like non-proteinogenic isovaline and α-aminoisobutyric acid that are vanishingly rare or absent from life forms on Earth, have been found in high abundance on non-terrestrial carbonaceous meteorites. It is therefore conceivable that exo-microorganisms might contain proteins that include these rare amino acids. We therefore asked whether the mammalian immune system would be able to recognize and induce appropriate immune responses to putative proteinaceous antigens that include these rare amino acids. To address this, we synthesised peptide antigens based on a backbone of ovalbumin and introduced isovaline and α-aminoisobutyric acid residues and demonstrated that these peptides can promote naïve OT-I cell activation and proliferation, but did so less efficiently than the canonical peptides. This is relevant to the biosecurity of missions that may retrieve samples from exoplanets and moons that have conditions that may be permissive for life, suggesting that accidental contamination and exposure to exo-microorganisms with such distinct proteomes might pose an immunological challenge.

## 1. Introduction

The search for exo-biosignatures and the characterization of exoplanetary atmospheres and surfaces is an objective of the NASA Astrobiology Program [1,2]. Life as we know it requires the availability of liquid water and therefore those planetary bodies outside Earth that have or may have had aqueous environments are of particular interest in the search for life beyond Earth. The dry river valleys on Mars are remnants of an oceanic history [3], and liquid water may still exist on Martian polar caps or below its surface [4]. Large oceans of liquid water are also believed to exist under the ice sheets of Jupiter’s moon Europa [5] and Saturn’s moon Enceladus [6]. This is evidenced by the Cassini mission’s observations of frozen water plumes at the south pole of Enceladus and the presence of oxygen, nitrogen, carbon, phosphorous, and sulphur, and the Hubble telescope’s images of similar cryogeyser activity on Europa. Estimates of the number of Earth-like planets orbiting Sun-like stars in our galaxy exceeds six billion [7,8,9] and spectroscopic signatures of water vapour has been reported in the atmosphere of exoplanets such as K2-18b [10].

Interestingly, chondrite meteorites contain up to 5%, mostly inorganic, carbon but a high proportion of organic compounds [11]. A number of amino acids, polyols, sugars, sugar alcohols, and sugar acids have been found on carbonaceous chondrite meteorites including the Murchison and Murray meteorites [12] and Tagish Lake meteorite [13]. The Murchison meteorite that fell in Australia in 1969 contains over 100 amino acids amongst many thousands of organic molecules ranging from two to nine carbons [14,15,16]. Although meteoritic amino acids could be formed in the parent bodies by the Strecker reaction, in which aldehyde or ketone reacts with cyanide and ammonia followed by hydrolysis to produce α-amino acid [17] but would produce only α-amino acids (amino and carboxyl group at the same carbon), and cannot explain the formation of β, γ and δ structures. Rigorous analytical methodologies excluded the possibility that this was due to contamination with organic molecules from Earth.

Of the organic molecules found in these meteorites, most are chiral, existing as both L- and D-stereoisomers (enantiomers). Terrestrial biology utilizes the L-enantiomer of amino acids preferentially but not exclusively [18] while abiotic processes typically produce racemic mixtures. In the Murchison meteorite, several amino acids including alanine and isovaline exist in non-racemic (L-excess) mixtures [19,20]. Although non-biotic explanations have been suggested for this homochirality, these observations have supported speculations about exo-biological signatures within the solar system [21].

Two reports have described polymers of amino acids in carbonaceous meteorites, the first being of di-glycine [22], and second large polymers of mainly glycine in the CV3 class carbonaceous chondrite Allende [23] before further characterization of amino acid polymers in Acfer 086 and Allende meteorites [23,24] identified the first protein in a meteorite hence in any extra-terrestrial source [25]. The amino acid signatures in the meteorites like Murchison and Tagish Lake meteorite [13] include the presence of high concentrations of non-protein amino acids that are exceedingly rare on Earth, such as isovaline and α-aminoisobutyric acid, and extremely low concentrations of amino acids that are common in terrestrial biota [19,26]. Those non-protein α-dialkyl-amino acids have been used as an indication of the indigeneity of meteoritic amino acids. These amino acids have also been detected in peptides produced by some filamentous fungi, suggesting the possibility of a terrestrial biotic source for some of the amino acids observed in meteorites. However, the relatively simple distribution of the C4 and C5 amino acids in fungal peptides (peptaibols or peptaibiotics) is distinct from the complex distribution observed in many carbonaceous chondrites, and extensive diagnostic analyses of stable isotope composition ruled out fungal contamination as a source of meteorite amino acids [27].

If microbial life evolved outside Earth, it is conceivable that the composition of such organisms may include such unusual but available organic molecules. Space missions such as the Mars 2020 seek evidence of exo-life and may attempt the retrieval of samples from planets or moons where life could exist. This poses potential biosecurity risks and problems of contamination of the Earth ecosystem and even infection by exo-microorganisms [28]. Here, we investigate the hypothetical risk of inefficient immune responses upon encountering organisms composed of antigens that could contain these rare exo-amino acids. We pose the question as to whether such exo-peptides have the capacity to be processed by cells of the mammalian immune system and induce adaptive immune responses.

To do this, we synthesised peptides in vitro based on a backbone of an immunodominant peptide epitope of ovalbumin that incorporated isovaline and α-aminoisobutyric acid residues at a range of positions. We then examined the efficiency of these peptides to activate and induce expansion of CD8^+^ T cells from ovalbumin-specific T cell receptor (TCR) transgenic OT-I mice.

The progression of T cells from a resting state to fully activated, proliferating cells is a crucial step in the initiation of an immune response. CD8^+^ T cells recognize pathogen-derived peptides of 8–10 amino acids in length that are bound to the major histocompatibility complex (MHC) class I receptor presented by antigen-presenting cells (APC) [29,30,31]. Naïve CD8^+^ T cells then undergo a program that drives them to expand and differentiate into cytotoxic effector cells that can kill and eventually clear the pathogen [32]. The capacity to proliferate following cognate antigen recognition is an important aspect of mammalian T cell immune response. In our work, we analyse CD8^+^ T cell activation and subsequent proliferation upon stimulation with exo- amino acid containing peptides. The results indicate that antigen recognition and immune response of mammalian cells occurred in response to the exo-peptides but that this was less efficient than for the canonical control peptide.

## 2. Materials and Methods

### 2.1. Synthesis of Peptides

Peptides were prepared through 9-Fluorenylmethoxycarbonyl (Fmoc) solid phase microwave assisted peptide synthesis (SPPS) using a Liberty Blue™ Automated Microwave Peptide Synthesizer (CEM). Coupling agents used a 1M *N,N*′-diisopropylcarbodiimide (DIC) solution in dimethylformamide (DMF) and 1 M ethyl cyano(hydroxyimino)acetate (Oxyma) pure as additive. Fmoc deprotection was performed by a 20% piperidine solution in DMF. 

The appropriate target sequences required for the immunological experiments were designed in such a way that the canonical peptide epitope was flanked by isovaline and α-aminoisobutyric acid. Peptides were based on a core sequence of the native ova amino acid sequence (OVA_257-264_). These residues (single amino acid code SIINFEKL) represent an antigenic epitope that is recognised by the transgenic TCR (T cell receptor) expressed by OT-I cells. This peptide is commonly used for studies of antigen-specific immune responses because CD8^+^ T cells from this mouse strain primarily recognize OVA_257-264_ when presented by the MHC I molecule [33].

For the synthesis of the peptides including the exo-amino acids (α-aminoisobutyric acid and isovaline), software was edited to include two new amino acids and the synthesis was performed according to a modified procedure (See supplementary info on peptide synthesis). Cleavage of the synthesised peptide from the resin and removal of side protecting groups was performed by treating the resin with a cleavage solution of 95% trifluoroacetic acid (TFA), 2.5% triisopropylsilane (TIS) and 2.5% of water for 3 h at room temperature. TFA was removed in a stream of air and the peptide precipitated by addition of cold diethyl ether.

Crude peptides were purified by preparative Reverse Phase High Performance Liquid Chromatography (RP-HPLC) using an Agilent 1260 system and a Phenomenex Jupiter C18 preparative column (5 µm. 300 Å, 21mm × 250 mm D × L) or a Phenomenex Luna C18(2) preparative column (5 µm, 100 Å, 21mm × 250 mm D × L) using an optimized gradient (Supplementary Figure S1).

Purity of the peptides was evaluated by HPLC-MS analysis using an Agilent 1200 HPLC equipped with a Diode Array Detector (DAD) and coupled with a single quadrupole mass detector using a Phenomenex Jupiter C18 analytical column (5 µm, 300 Å, 4.6 mm × 150 mm D × L) or a Phenomenex Luna C18 analytical column (5 µm. 100 Å, 4.6 mm × 250 mm D × L) and the appropriate eluent gradient. (Supplementary Figure S2). Peptides were generally >98% pure except the long native peptide that was 93% pure and was shown to be immunologically active.

### 2.2. Assays and Reagents

Ultra-pure lipopolysaccharide (LPS) from *Escherichia coli* 0111:B4 was purchased from InvivoGen. The following antibodies were purchased from BD Bioscience: APC (Allophycocyanin) Rat Anti-Mouse CD8a (Catalog No. 553035), PE Rat Anti-Mouse CD25 (Catalog No. 553866), PerCP-Cy™5.5 (Peridinin-chlorophyll proteins cyanine) Hamster Anti-Mouse CD69 (Catalog No. 551113) and eBioscience™ Fixable Viability Dye eFluor™ 780 (Catalog No. 65-0865-14), OneComp eBeads™ Compensation Beads (Catalog No. 01-1111-42), CellTrace™ Violet Cell Proliferation Kit (Catalog No. C34571) were purchased from Thermo Fisher Scientific/Invitrogen. BD FACS™ lysing solution for red blood cell lyses was purchased from Becton, Dickinson and Company, BD (Catalog No. 555899), Fmoc-L-α-aminoisobutyric acid was purchased from Sigma (Fmoc-L-Aib-OH; CAS 94744-50-0) and Fmoc-L-isovaline (CAS 857478-30-9) from Hangzhou MolCore BioPharmatech Co., China.

### 2.3. Splenocytes Isolation and T Cell Response

Female OT-I mice that express a T cell receptor (TCR) that is specific for an ovalbumin (OVA) peptide presented by the MHC class I molecule H2-K^b^ were purchased from Charles River, UK. These mice contain the transgenic T cell receptor (insertion of Tcra-V2 and Tcrb-V5 genes) that is designed to recognize ovalbumin peptide residues 257–264 (OVA_257-264_) in the context of H2K^b^ (CD8 co-receptor interaction with MHC class I). This results in MHC class I-restricted, ovalbumin-specific, CD8^+^ T cells (OT-I cells). Because the CD8 T cells of this mouse primarily recognize OVA_257-264_ when presented by the MHC I molecule, it allows the study of immune response dynamics of CD8^+^ T cells [33]. Mice were provided with food and water ad libitum. Animal experiments were conformed to the animal care and welfare protocols approved by UK Home Office (project license P79B6F297) in compliance with all relevant local ethical regulations. For the in vitro experiments, mice were culled humanely and their spleens collected under sterile conditions. A uniform single cell suspensions from OT-I mice spleens were generated by gently meshing the tissue through a 100 µm pre-sterilized cell strainer (Thermo Fischer Scientific) and suspended directly into the cold Roswell Park Memorial Institute (RPMI) 1640 media. Red blood cells were lysed with 1x lysis buffer (Catalog No. 555899 from Becton, Dickinson and Company, BD) and the remaining cells were collected following low speed spins (300 g, 5 min, 8 °C) and then resuspended in pre-warmed complete RPMI 1640 medium (Dutch modification) supplemented with 10% heat-inactivated fetal bovine serum (FBS), 100 U/mL penicillin and 100 mg/mL streptomycin, 1% HEPES+ 50 µM 2-mercaptoethanol. The cell density was adjusted to 1 × 10^7^ cells/mL using a hemocytometer counting-chamber before pre-labelling with CellTrace Violet dye (1: 1000 diluted from Invitrogen) for 20 min at 37 °C. Cells were then seeded in 96 well round-bottom plates at 1 × 10^6^ cells/well in 100 μL complete medium supplemented with 50 ng/mL ultra-pure lipopolysaccharide (LPS) from *Escherichia coli* 0111:B4 (InvivoGen) and peptide antigens. OT-I cells were stimulated with 100 nM of the peptides. Cells were incubated for 24, 48 and 72 h in a CO_2_ incubator (5% CO_2_) at 37 °C. The cell activation state was determined by measuring the expression of the high-affinity IL-2 receptor (CD25) over 3 days. T cell proliferation was determined by assessing the dilution of the CellTrace Violet fluorescence dye following incubation of pre-labelled naïve T cells with the tested peptides after 72 h. Three technical replicas were performed in each experiment. T cell activation and T cell proliferation experiments were performed three times to determine the percentage of T cell activation, T cell proliferation and the mean florescent intensity (MFI) of proliferating activated (CD25 expressing) T cells. Statistical differences between the means of two data set (student *t*-test) were determined with GraphPad Prism scientific 2D graphing and statistics software.

### 2.4. Flow Cytometric Analyses

Flow cytometry (FCM) has been applied in this study to quantify the distribution, proliferation and activation status of the CD8 T cells. Flow cytometry allows the simultaneous measurement of multiple physical and chemical parameters of a populations of cells. Flow cytometry data analysis based on ‘gating’ of subpopulations of cells quantified on their relative expression of different fluorescent markers. The gating strategy is shown in Supplementary Figure S3. For the analysis, FlowJo cytometry data analysis software (BD Bioscience) was used and the significance of the difference between the means of two data set (student *t*-test) was determined with GraphPad Prism scientific 2D graphing and statistics software.

Immunophenotyping of specific cell types used different fluorochrome conjugated antibodies against specific receptor on the cell surface. The antibodies including the fluorochromes used in this study are listed above in the part assays and reagents. For this purpose, cell surface staining with the appropriate antibody-fluorophore conjugates (0.2 mg/mL) was performed in staining buffer (PBS containing 2% (*v*/*v*) fetal calf serum and 2 mM EDTA), for 30 min at 4 °C. Cells were stained with anti-CD8 antibody to identify CD8 T cells and the anti-CD25 and anti-CD69 antibodies were used to evaluate the activation state of the cells. Activated lymphocytes were analysed for cell proliferation by measuring dilution of CellTrace Violet dye (450nm_EX_/450nm_EM_, Thermo Fisher Scientific, Waltham, MA, USA). The acquisition of the samples was performed using the benchtop analyser Attune NxT Acoustic Flow Cytometer (Thermo Fisher Scientific).

## 3. Results

### 3.1. Peptide Synthesis to Generate High Purity Ova-Exo-Peptides

**Figure 1:**
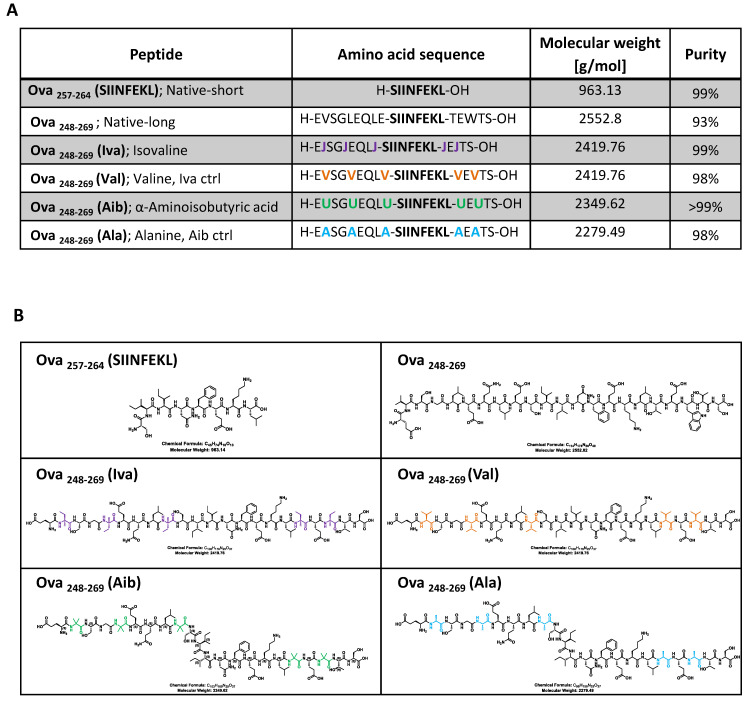
Peptides used in this study. Peptides were synthesized by microwave assisted solid-phase synthesis using a CEM Liberty Blue peptide synthesiser. (**A**) Crude peptides were purified by Preparative HPLC (Supplementary Figure S1), identity and purity was evaluated by HPLC-MS (Supplementary Figure S2). Peptides synthesised used in this study based on ovalbumin (Ova), the main protein in egg white widely used as an antigen for immunization research. The specific amino acid residues Ova_257-264_ (SIINFEKL) represent a T cell-dependent antigen used as a model peptide epitope for studying antigen-specific immune responses in mice. The position of amino acid exchange within the ova peptide, encompassing the core-SIINFEKL, is indicated by coloration; (**B**) the chemical structure of the synthesised peptide chains. The colored amino acids match the color code in A showing the position of the amino acids within the peptide structure.

Peptides were synthesized by solid phase microwave assisted peptide synthesis (SPPS) using a Liberty Blue™ Automated Microwave Peptide Synthesizer. Peptide sequence based on the short hen ovalbumin backbone “SIINFEKL” that included isovaline and α-aminoisobutyric acid (Figure 1A,B). To assess antigen processing by antigen presenting cells, the immunodominant peptide epitope “SIINFEKL” was extended; Ova_(248-269)_ EVSGLEQLE-SIINFEKL-TEWTS with control or exo-amino acids flanking the Ova_(257-264)_ SIINFEKL epitope. The positions of the amino acids within the longer Ova peptide (Ova_248-269_) are color-coded in Figure 1A,B. The peptides were purified by RP-preparative HPLC, and purity and identity of the purified peptides were assessed by HPLC-MS (Figure 1A).

The synthesised peptides that harbour non-canonical amino acids were: Ova_248-269_ (Iva) that contained the amino acid isovaline and Ova_248-269_ (Aib) that contained the amino acid α-aminoisobutyric acid. Control peptides were synthesised with canonical amino acids at the same positions as Iva and Aib: Ova_248-269_ (Val) that contained valine as a control for isovaline and Ova_248-269_ (Ala) that contained alanine as a control for α-aminoisobutyric acid. The ova peptide, native-long, (Ova_248-269_) consisted of the native ovalbumin amino acids and served as the positive control in antigen processing assays.

Peptides were generally >98% pure except the long native peptide that was 93% pure (Figure 1A). Figure 1B shows the chemical structure of the synthesised peptide chains using the same color code as shown in the amino acid sequence in Figure 1A to highlight the exact position of the amino acids within the chemical peptide structure.

T cell activation and proliferation assays based on SIINFEKL peptide, which is presented by MHC class I receptors on the surface of antigen-presenting cells (APC), are flanked in the exo-peptides by Iva and Aib or in the canonical peptide controls with valine or alanine. The presentation of the eight amino acids “SIINFEKL” within any of the extended peptides therefore requires processing of the peptides, obtained by the proteasome and by endo- and exo-peptidases that liberate the immunogenic peptide “SIINFEKL” from the extended peptide sequence. The release of the peptide from proteins involves C-terminal proteasomal cleavage, while the generation of the processed *N*-terminus involves the action of bespoke peptidases [34]. The short ova-peptide “SIINFEKL” (native short, shown in Figure 1A,B) served as a positive control for successful MHC class I antigen presentation and T cell activation [35]. The longer peptide Ova_(248-269)_ EVSGLEQLE-SIINFEKL-TEWTS (native long, shown in Figure 1A,B) consists of the native amino acid sequence of the ovalbumin peptide and served as a control for successful APC antigen processing following T cell activation.

### 3.2. Iva- or Aib Containing Peptides Activate OT-I T Cells

The progression of CD8 T cells from a resting state to fully activated is a crucial step in the initiation of an immune response.

T cell stimulation assays were carried out in the presence of modified Ova-peptides and OT-I cell activation responses (CD25 expression) were monitored. CD25 is the α-chain of the Interleukin-2 (IL-2) receptor and is expressed only on activated T cells [36] and is commonly used as a marker for activated T cells. In the absence of an adjuvant, splenocytes from naïve mice fail to drive activation of OT-I cells. Therefore, LPS was added to cultures to prime antigen presenting cells before adding the modified peptides.

**Figure 2:**
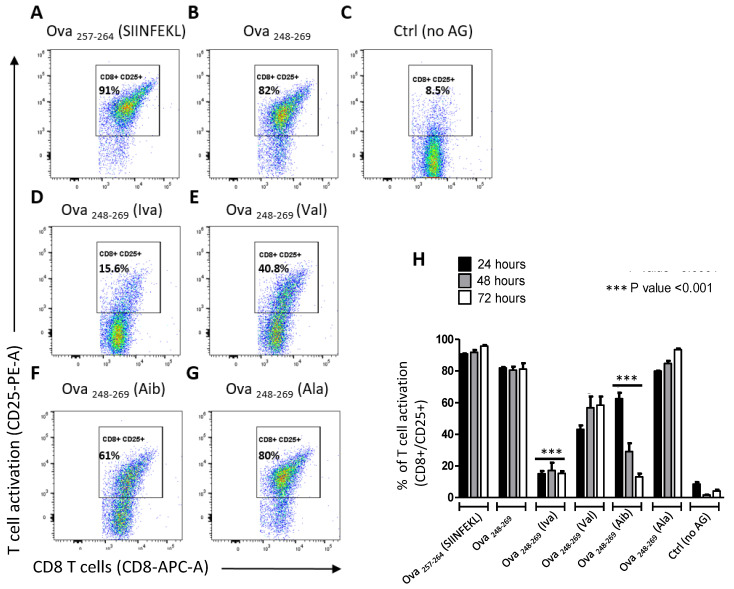
Iva- or Aib containing peptides activate T cells. Cell surface expression of CD25 molecules on OT-I cells following stimulation with peptide antigens. (**A**–**G**) representative flow cytometry dot plots show CD8 T cell activation after 24 h stimulated with either the native ova-control peptides ((**A**): short Ova_257-264_ (SIINFEKL) or (**B**): longer Ova_248-269_), unstimulated (**C**) or with the ova peptides substituted with exo amino acids (**D**): Ova_248-269_ (Iva) and (**F**): Ova_248-269_ (Aib) or peptides substituted natural amino acids (**E**): Ova_248-269_ (Val) and (**G**): Ova_248-269_ (Ala); (**H**) frequency of CD25 positive single CD8 cells from three technical replicas of three biological replicas. Activation of Iva-peptides and Aib-peptide was significantly reduced compared to the native Ova peptide control ((**B**,**H**) *p*-value < 0.0001) or the peptides substituted with Val ((**E**,**H**) 24 h and 72 h: *p*-value < 0.0001; 48 h: *p*-value 0.0003) or Ala ((**G**,**H**) 24 h: *p*-value 0.0009; 48 h and 72 h: *p*-value < 0.0001).

Cultures activated with SIINFEKL peptide and long OVA peptide had increased frequency of activated OT-I cells (Figure 2A,B) relative to non-activated T cells (Figure 2C) and (Figure 2H). The modified peptides all induced CD25 expression as early as 24 h following incubation with cells; however, the frequency of CD25^+^ OT-I cells remained low for the 72 h duration of the experiment when treated with peptide Iva (~15% CD25+ cells). In comparison, peptide Aib induced a unique kinetic response with declining frequency of activated cells over the 72 h incubation period (~60% to ~15% CD25+ cells) relative to control peptides (Figure 2H). In addition, similar to CD25, analysis of the early activation marker, CD69, was found to be induced when OT-I cells were stimulated with the exo-peptides (Supplementary Figure S4).

### 3.3. Iva- or Aib Containing Peptides Induce OT-I Cell Proliferation

**Figure 3:**
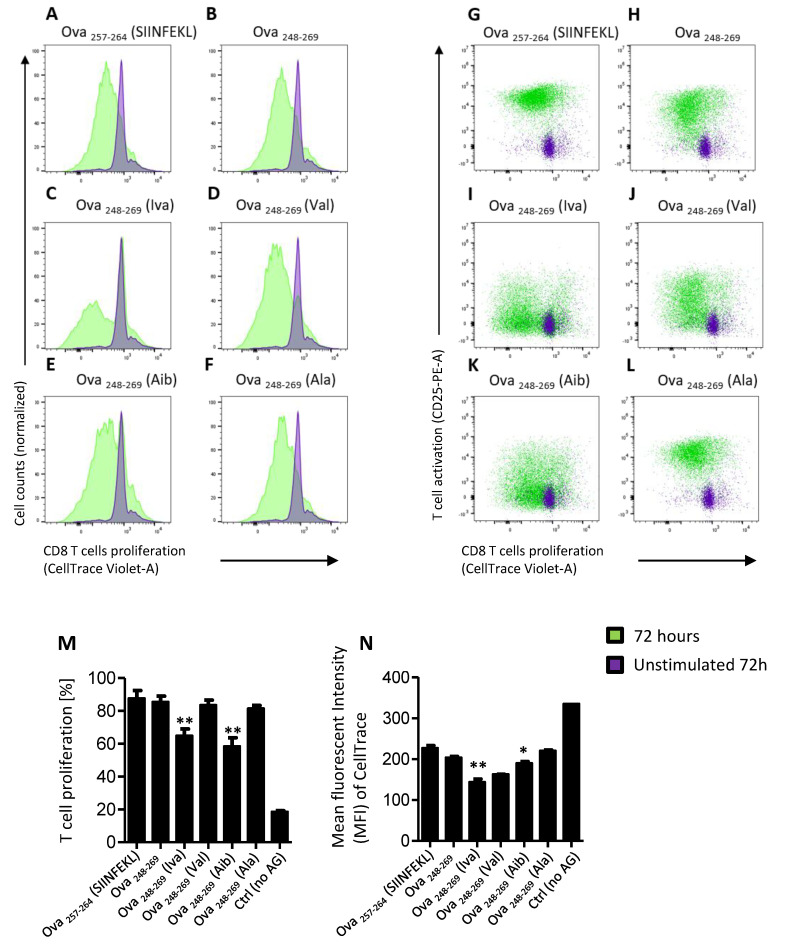
Iva- or Aib promote proliferation of OT-I cells. Cell proliferation was assessed by measuring dilution of CellTrace Violet dye over 72 h following stimulation with the test peptides. (**A**–**L**) Representative images show a histogram shift (**A**–**F**) of unstimulated control cells (purple histogram) against cells stimulated with peptides (green). (**G**–**L**): Representative images show dot plots of activated (CD25 expressing) cells proliferating (decrease in CellTrace) cells when unstimulated (purple) or stimulated with peptides (green). The native ova-control peptides (**A**,**G**): short Ova_257-264_ (SIINFEKL) or (**B**,**H**): longer Ova_248-269_). Ova peptides substituted with exo-amino acids (**C**,**I**): Ova_248-269_ (Iva) and (**E**,**K**): Ova_248-269_ (Aib) or peptides substituted natural amino acids (**D**,**J**): Ova_248-269_ (Val) and (**F**,**L**): Ova_248-269_ (Ala). (**M**): Geometric Mean (CellTrace) of proliferating CD8 T cells. Proliferation with peptide Ova_248-269_ (Iva) or Ova_248-269_ (Aib) are statistical significant (*p*-value < 0.01) compared to the control peptides Ova_248-269_ (*p*-value 0.0032 and *p*-value 0.0015) and control Ova_248-269_ (Val) and Ova_248-269_ (Ala), *p*-value 0.004 and *p*-value 0.0021, respectively. (**N**): Mean fluorescent Intensity (MFI) of proliferating CD8 T cells expressing CD25. Peptide Ova_248-269_ (Iva) or Ova_248-269_ (Aib) are statistical significant compared to the control peptides Ova_248-269_ (**: *p*-value < 0.01; *: *p*-value < 0.1). Three technical replicas were performed for each of the three biological replicas.

Capacity to proliferate following cognate antigen recognition is an important aspect of mammalian T cell immune response. We determined proliferation of cells following incubation of pre-labelled naïve T cell with the tested peptides at 24 h, 48 h (Supplementary Figure S5) and 72 h. Iva and Aib peptides supported expansion of OT-I cells (Figure 3C,I,E,K, respectively, and Figure 3M) with frequency of proliferating cells reaching ~60% of the population, compared to over 80% in control peptides (Figure 3A,G (SIINFEKL); Figure 3B,H (OVA_(248-269)_); Figure 3D,J (Val); Figure 3F,L (Val) and Figure 3M. In addition, the rate of proliferation appeared to be similar in all tested peptides except for peptide Iva and the control peptide Val (Figure 3N) that showed lower CellTrace MFI values.

## 4. Discussion

In this paper, we consider whether the immune system is able to recognise peptides containing amino acids that are not commonly found in terrestrial organisms but are known to be common in meteorites, and therefore may be represented in non-terrestrial life forms. It is likely that NASA or commercial space exploration by companies such as SpaceX, Virgin Galactic or Blue Origin will promote travel and exploration of other planets and the sending of long-range probes to retrieve samples. Such missions could contaminate exo-environments with terrestrial microorganisms that temporarily survived some of the harsh conditions of space-radiation, vacuum, extremely variable temperatures, etc., [37,38] but could also encounter non-terrestrial microorganisms that may have evolved in aqueous environments that have the potential to colonize or even infect humans and other animals. Liquid water is likely to exist on Jupiter’s moon Europa and on Saturn’s moon Enceladus and may exist below the surface of Mars [5,6]. Experiments performed in NASA’s Viking mission inoculated Martian soil with ^14^C-labeled nutrients in water, but failed to provide conclusive evidence of extant life, although the release of ^14^CO_2_ from these samples was intriguing [39].

On Earth, the boundary conditions under which life can exist has shown that microbial life is possible even at extremes of temperature, pH, pressure, radiation, salinity, energy, and nutrient limitation, as long as there is liquid water. Extremophiles, which span all three domains of life: bacteria, archaea and eukaryotes are important because of their enormous biotechnology potential but also because what they can teach us about the fundamentals of biochemical and structural biodiversity. Extremophile research reinforces the suggestion that microbial life may exist on other planetary and celestial bodies [40] demonstrated by several studies that showed the growth of earth microorganisms under lab-simulated planetary conditions, including Mars-like [41,42,43] and Enceladus-like [44] conditions.

A recent review proposed two hypothetical scenarios of the human immune system interacting with alien microorganisms-terrestrial microbes that have grown and adapted to an alien environment or alien exo-microorganisms with different biochemistries and antigenicity profiles [28]. Both settings anticipate the possible encounter of the terrestrial immune defense systems with distinct or novel organisms and biomolecules that may have radical departures from terrestrial canonical signatures that activate immune responses.

The induction of innate and adaptive immune responses towards invasive microorganisms is crucial for defense. We tested putative “exo-peptides” with antigenic potential that included amino acid substitutions with isovaline (Iva) and α-aminoisobutyric acid (Aib) that are present abundantly in carbonaceous meteorites [14]. This anticipates a potential hypothetical encounter of the immune defense system with an antigen that contains those rare amino acids. We tested whether these non-canonical amino acids affected antigen processing, presentation, and stimulation of T cell responses of the model ovalbumin SIINFEKL epitope. We showed that the consequences of MHC class I antigen presentation in terms of T cell induction and proliferation was less efficient when presented with these exo-peptides than with canonical peptide controls.

This has implications for astronauts and space exploration missions designed to retrieve samples from potentially biotic exo-environments. The effects of exposure to a novel microbe could be exacerbated in astronauts where the human body and the immune system have already been exposed to sustained extreme conditions and environmental stress [45]. Although astronauts have been shown to be able to survive in good health after many months in space, there is evidence that space flight can progressively weaken immune responses [46]. Human neutrophils and monocytes post space flight exhibit reduced capacities of bacteria phagocytosis and an attenuated oxidative burst and degranulation [47,48]. Decreased responsiveness for host defense cells against potential invading pathogens could have contributed to the observation that nearly half of all Apollo crew members suffered from microbial infections shortly after they returned from space missions. Reduced T cell activation following proliferation after exo-peptide stimulation in our study suggests a risk that the potency of protective immune surveillance mechanisms could be attenuated when challenged by novel antigenic signatures.

It is not clear whether an extraterrestrial microorganism adapted to a non-terrestrial, extreme environment would be pathogenic in a human host. Similarly, it is likely that they would be poorly adapted to the conditions of the human body, and their capacity to colonize and infect us would be limited. However, a thermophilic bacterium *Mycobacterium xenopi* was found in a hospital’s hot water system and three out of 87 patients exposed to this microorganism developed pulmonary mycobacteriosis [49] suggesting that some terrestrial extremophiles have pathogenic potential for humans. Gene expression of *Enterobacter bugandensis* isolated from the International Space Station (ISS) showed an increase in expression of genes involved in antimicrobial resistance (AMR), multiple drug resistance (MDR) and genes related to virulence and disease [50]. Additionally, the competency of bacteria to acquire foreign genetic material was observed to increase in microgravity [50]. This number of changes to the pathogenicity of microbes might become relevant during prolonged space travel. Even if the pathogenic potential for exo-microorganisms was inherently attenuated, it is also possible that they could induce allergic reactions or create novel toxigenic compounds [28].

We show that exo-peptides were recognized by the mammalian immune system, but that the strength of the immune response was decreased. We can anticipate that the conditions of space travel impact on the immune system [45]. Astronauts on extended spaceflights experienced already a general decay in T cell function, accompanied by persistent reductions in production of cytokines such as interleukin (IL)-5, IL-6, IL-10, interferon gamma (IFNγ), and tumor necrosis factor alpha (TNFα) [51]. In addition, studies on animals had shown that space flight not only affect cytokine production [52] and leukocyte subpopulation distribution of T lymphocytes (CD8^+^ T lymphocytes and interleukin-2 receptor-bearing T lymphocytes) [53], but also severe inhibition of bone marrow response to the colony-stimulating factors have been recorded in experimental rats and monkeys [52,53,54]. Both the altered leukocyte distribution and impaired ability of precursor cells to differentiate into mature, immunologically competent cells contribute to alterations in cell-mediated immunity and impaired immune function observed after space flight.

Naïve CD8^+^ T cells play a key role in protective immunity in response to foreign by differentiation into cytotoxic effector cells [32]. At peak response, these effector T cells secrete high amounts of cytokines [interferon-gamma (IFNγ) and tumor necrosis factor alpha (TNFα)] and cytolytic molecules (granzymes and perforin). Subsequently, after elimination of the antigenic source, most of these effector T cells undergo apoptosis, and a few survive and become central memory and effector memory T cells [55,56]. This differentiation process is tightly controlled and changes in the nature, context and duration of antigen exposure can alter the process leading to T cell dysfunction, unresponsiveness and/or death. The experiments outlined here suggest that multiple elements of the vital roles of T cell mediated protection could be attenuated when responding to non-canonical antigens.

In conclusion, the amino acids isovaline (Iva) and α-aminoisobutyric acid (Aib) have been identified as common organic molecules on chondrites. We show that the mammalian immune system recognized peptides containing these exo-amino acids but that T cell activation and proliferation was reduced. Future studies should extent our studies to encompass the immunomodulatory effects of a wider range of organic components including exo-sugars and other novel organics molecules that have been found on meteorites.

## 5. Implications

This is one of the first studies in the field of “exo-immunology” that we recently proposed to investigate the interaction of the immune system of terrestrial organisms with alien microbes or terrestrial microorganisms that have changed under the influence of space environments [28]. Our initial finding speculates that, if microbial life is subsequently discovered outside Earth, and the protein content of their cells includes rare organic molecules found in meteorites, then they could pose an immunological challenge to humans and other animals. We therefore propose that space explorations that intend retrieving samples from aqueous environments in our solar system should acknowledge and mitigate the possible immunological threats posed by accidental exposure to novel exo-microorganisms.

## Supplementary Materials

The following are available online at https://www.mdpi.com/2076-2607/8/7/1066/s1.
