# Supplementary material for: A Weakened Immune Response to Synthetic Exo-Peptides Predicts a Potential Biosecurity Risk in the Retrieval of Exo-Microorganisms
Source: Microorganisms. 2020 Jul 17;8(7):1066. doi: 10.3390/microorganisms8071066 (PMC7409182; doi:10.3390/microorganisms8071066)
Supplement: Supplementary file 1 [file microorganisms-08-01066-s001.pdf]

**A**

Ova$_{257-264}$ ; Native-short
H-**SIINFEKL**-OH
**R$_t$ =6.85 min**

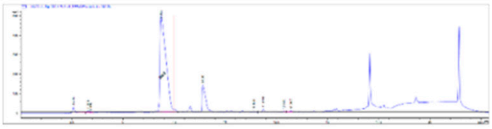

**B**

Ova$_{248-269}$ ; Native-long
H-EVSGLEQLE-**SIINFEKL**-TEWTS-OH
**R$_t$ = 12.08 min**

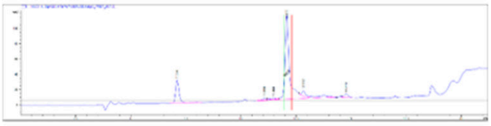

**C**

Ova$_{248-269}$ (Iva); Isovaline [J]
H-E**J**SG**J**EQL**J**-**SIINFEKL**-**J**E**J**TS-OH
**R$_t$ = 14.9 min**

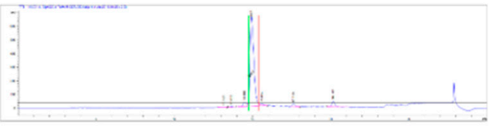

**D**

Ova $_{248-269}$ (Val); Valine [V]
Iva ctrl - H-E**V**SG**V**EQL**V**-**SIINFEKL**-**V**E**V**TS-OH
**R$_t$ = 12.31 min**

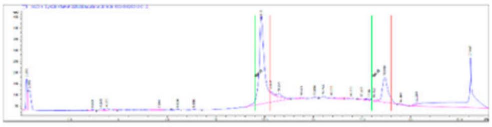

**E**

Ova $_{248-269}$ (Aib); α-Aminoisobutyric acid [U]
H-E**U**SG**U**EQL**U**-**SIINFEKL**-**U**E**U**TS-OH
**R$_t$ = 13.23 min**

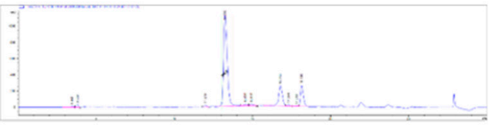

**F**

Ova $_{248-269}$ (Ala); Alanine [A]
Aib ctrl - H-E**A**SG**A**EQL**A**-**SIINFEKL**-**A**E**A**TS-OH
**R$_t$ = 11.27 min**

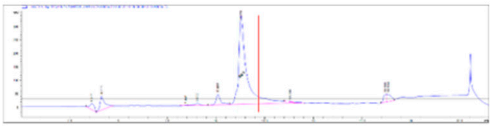

**Supplementary Figure 1. Preparative RP-HPLC methods and traces.** Crude peptides were purified by Preparative RP-HPLC using an Agilent 1260 system and A: Phenomenex Luna C18(2) preparative column (5 µm. 100 Å, 21 mm x 250 mm D x L) or B-F: Phenomenex Jupiter C18 preparative column (5 µm. 300 Å, 21 mm x 250 mm D x L) using an optimised gradient.

**A**

Ova$_{257\text{-}264}$ ; Native-short - H-**SIINFEKL**-OH

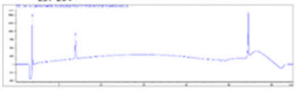

**UV 210 nm - $R_t$ =13.9 min - purity 99%**

MS spectrum of peak at 13.9 min

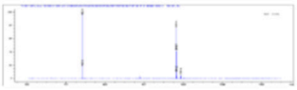

Mass calc. for $C_{45}H_{75}N_{10}O_{13}$ $[M+H]^+$ = 963.54, measured 963.5

Mass calc. for $C_{45}H_{76}N_{10}O_{13}/2$ $[M+2H]^{2+}/2$ = 965.56/2 = 482.78, measured 482.4

**B**

Ova$_{248\text{-}269}$ ; Native-long - H-EVSGLEQLE-**SIINFEKL**-TEWTS-OH

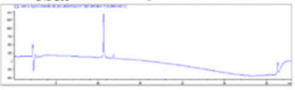

**UV 210 nm - $R_t$ =10.6 min - purity 93%**

MS spectrum of peak at 10.63 min

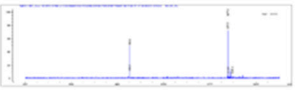

MW calc. for $C_{114}H_{179}N_{26}O_{40}/2$ $[M+2H]^{2+}/2$ = 2553.83/2 = 1276.9, measured 1277.3

MW calc. for $C_{114}H_{180}N_{26}O_{40}/3$ $[M+3H]^{3+}/3$ = 2554.83/3 = 851.61, measured 852.0

MW calc. for $C_{114}H_{179}N_{26}O_{40}$ = 2552.82, deconvoluted mass = 2552.78

**C**

Ova$_{248\text{-}269}$ (Iva); Isovaline [J]

H-EJSGJEQLJ-**SIINFEKL**-JEJTS-OH

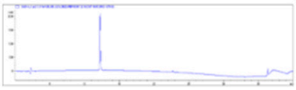

**UV 210 nm - $R_t$ =12.2 min - purity 99%**

MS spectrum of peak at 12.2 min

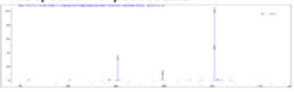

MW calc. for $C_{114}H_{179}N_{26}O_{40}/2$ $[M+2H]^{2+}/2$ = 2420.77/2 = 1210.38, measured 1210.8

MW calc. for $C_{114}H_{180}N_{26}O_{40}/3$ $[M+3H]^{3+}/3$ = 2421.77/3 = 807.25, measured 807.3

MW calc. for $C_{114}H_{179}N_{26}O_{40}$ = 2419.76, deconvoluted mass = 2419.23

**D**

Ova $_{248\text{-}269}$ (Val); Valine [V]

Iva ctrl - H-EVSGVEQLV-**SIINFEKL**-VEVTS-OH

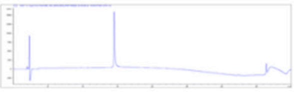

**UV 210 nm - $R_t$ =14.5 min - purity 98%**

MS spectrum of peak at 14.5 min

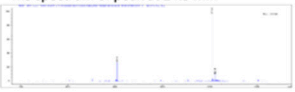

MW calc. for $C_{114}H_{179}N_{26}O_{40}/2$ $[M+2H]^{2+}/2$ = 2420.77/2 = 1210.38, measured 1210.8

MW calc. for $C_{114}H_{180}N_{26}O_{40}/3$ $[M+3H]^{3+}/3$ = 2421.77/3 = 807.25, measured 807.3

MW calc. for $C_{114}H_{179}N_{26}O_{40}$ = 2419.76, deconvoluted mass = 2419.23

**E**

Ova $_{248\text{-}269}$ (Aib); α-Aminoisobutyric acid [U]

H-EUSGUEQLU-**SIINFEKL**-UEUTS-OH

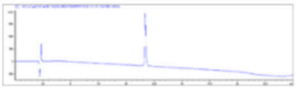

**UV 210 nm - $R_t$ =15.3 min - purity 99%**

MS spectrum of peak at 15.3 min

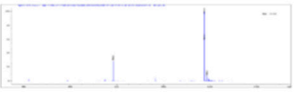

MW calc. for $C_{114}H_{179}N_{26}O_{40}/2$ $[M+2H]^{2+}/2$ = 2350.63/2 = 1175.32, measured 1175.7

MW calc. for $C_{114}H_{180}N_{26}O_{40}/3$ $[M+3H]^{3+}/3$ = 2351.64/3 = 783.88, measured 784.2

MW calc. for $C_{114}H_{179}N_{26}O_{40}$ = 2349.62, deconvoluted mass = 2349.48

**F**

Ova $_{248\text{-}269}$ (Ala); Alanine,

Aib ctrl - H-EASGAEQLA-**SIINFEKL**-AEATS-OH

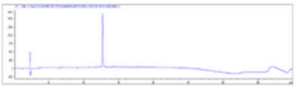

**UV 210 nm - $R_t$ =15.3 min - purity 98%**

MS spectrum of peak at 15.3 min

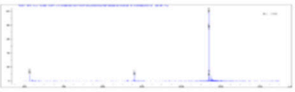

MW cal. for $C_{114}H_{179}N_{26}O_{40}/2$ $[M+2H]^{2+}/2$ = 2281.51/2 = 1140.76, measured 1140.3

MW calc. for $C_{114}H_{180}N_{26}O_{40}/3$ $[M+3H]^{3+}/3$ = 2282.52/3 = 760.84, measured 760.7

MW calc. $C_{114}H_{179}N_{26}O_{40}$ = 2279.49, deconvoluted mass = 2278.83

**Supplementary Figure 2. Analytical RP-HPLC-MS traces.** Purity of the peptides was evaluated by HPLC-MS analysis using an Agilent 1200 HPLC equipped with a Diode Array Detector (DAD) and coupled with a single quadrupole mass detector using A: Phenomenex Luna C18 analytical column (5 µm. 100 Å, 4.6 mm x 250 mm D x L) or B-F: Phenomenex Jupiter C18 analytical column (5 µm. 300 Å, 4.6 mm x 150 mm D x L) and the appropriate eluent gradient..

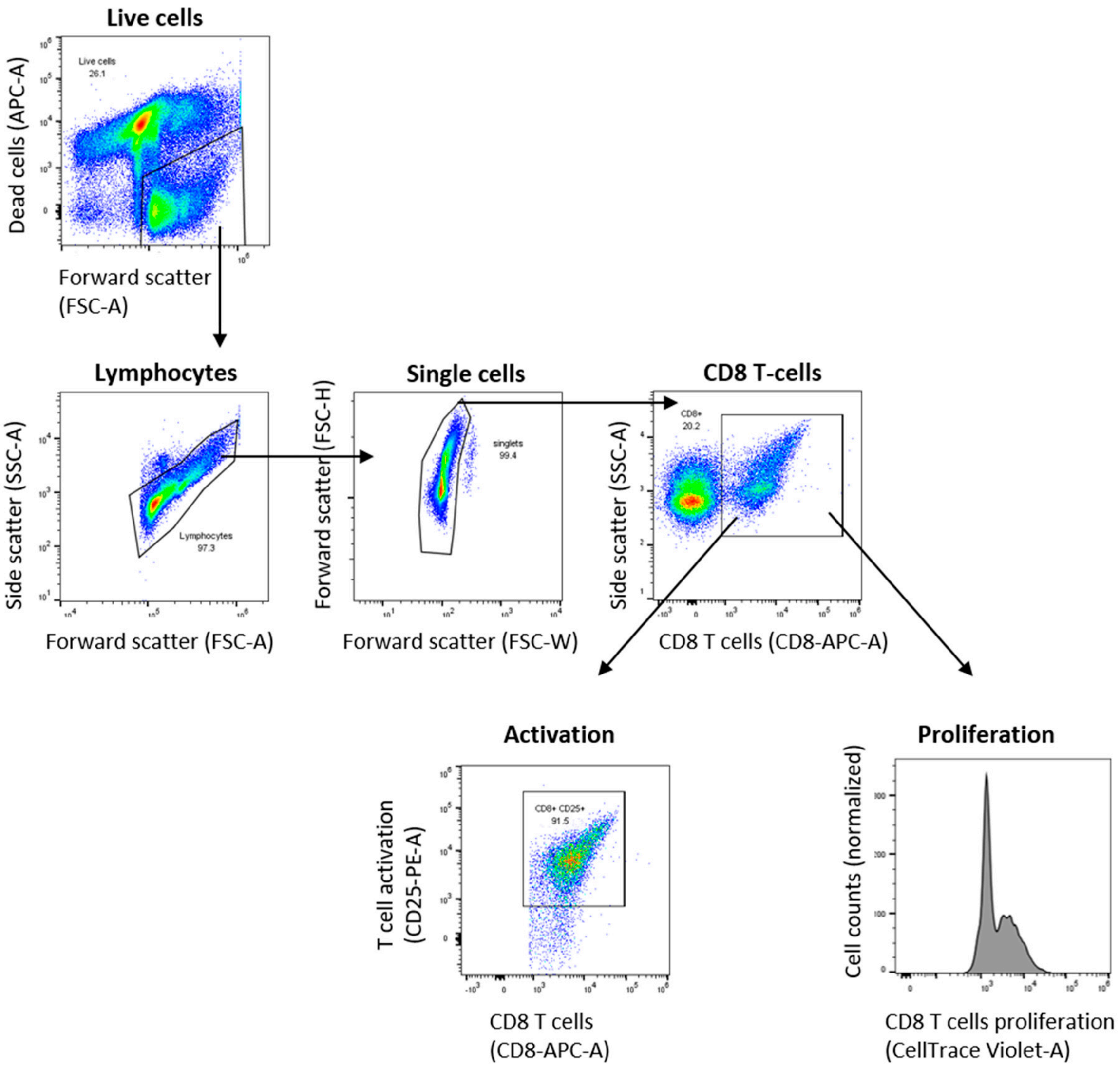


**Supplementary Figure 3: Flow cytometry gating pathway for T cell activation and proliferation markers.** Plots are showing the relationship of different fluorescence intensity of T cells labeled with antibodies against specific markers, measured in arbitrary units in log scale (see method section for details). Initial gating was set on live cells before lymphocytes were isolated using SSC-A vs FSC-A. After elimination of doublets the gate was set on CD8 T cells using the SSC-A vs CD8-APC-A. For T cell activation, a gate was set within CD8 T cells that expresses the cell surface marker CD25 ($CD8^+/CD25^+$). For cell proliferation, histograms were created using the number of $CD8^+$ T cells against the (reduction over time of) cell proliferation staining (CellTrace Violet).

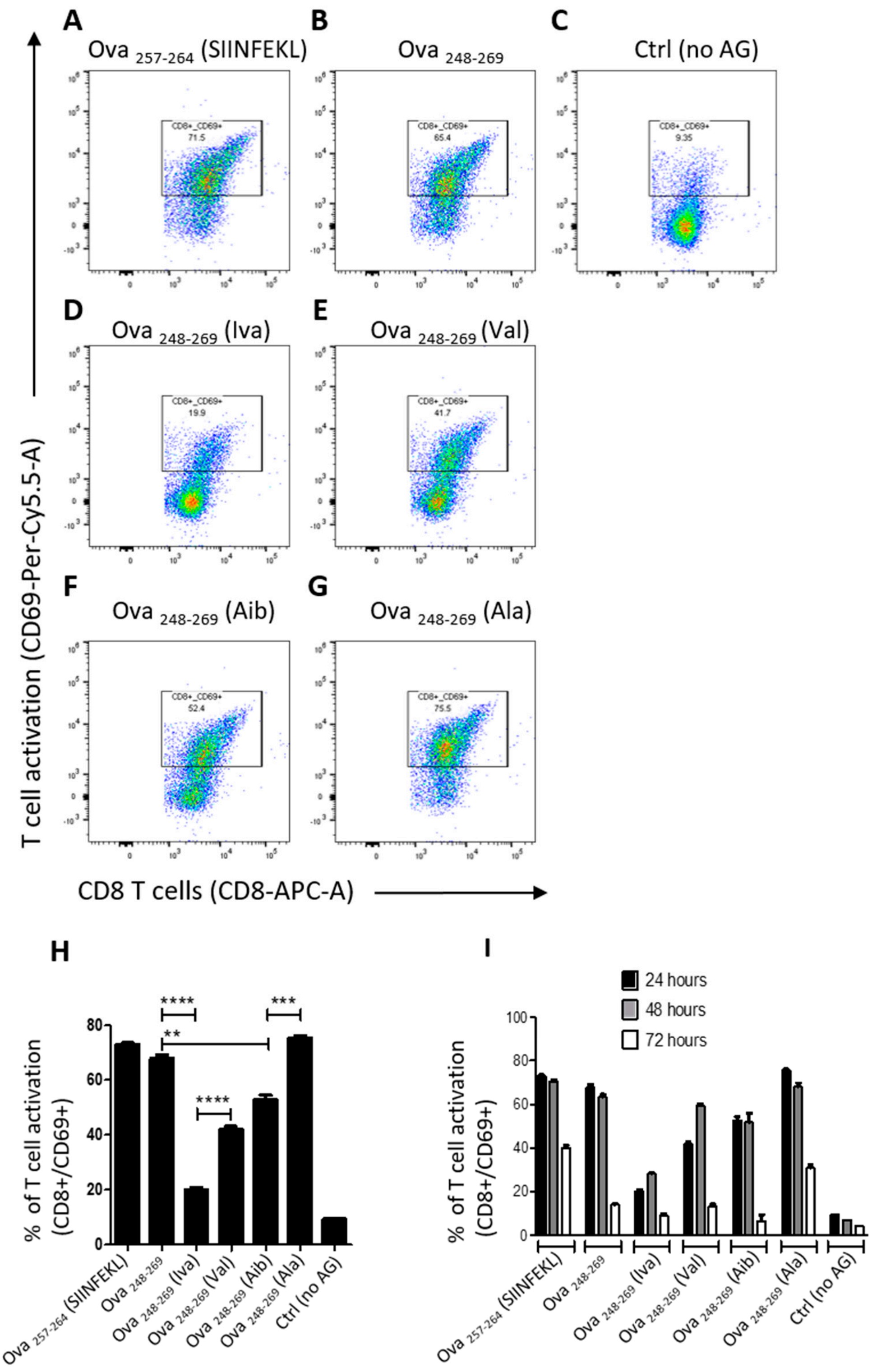


**Supplementary Figure 4: Assessment of cell activation via measuring the expression of the surface marker CD69 on CD8 T cells after stimulation with Ova-derived peptide epitopes.** A-G: Representative flow cytometry dot plots show CD8 T cell activation after 24 h stimulated with indicated peptides. H: Frequency of CD69 positive single CD8 cells after 24 h I: Frequency of CD69 positive single CD8 cells over time. Statistical significance (****: P value <0.0001; ***: P value <0.001; **: P value <0.01).

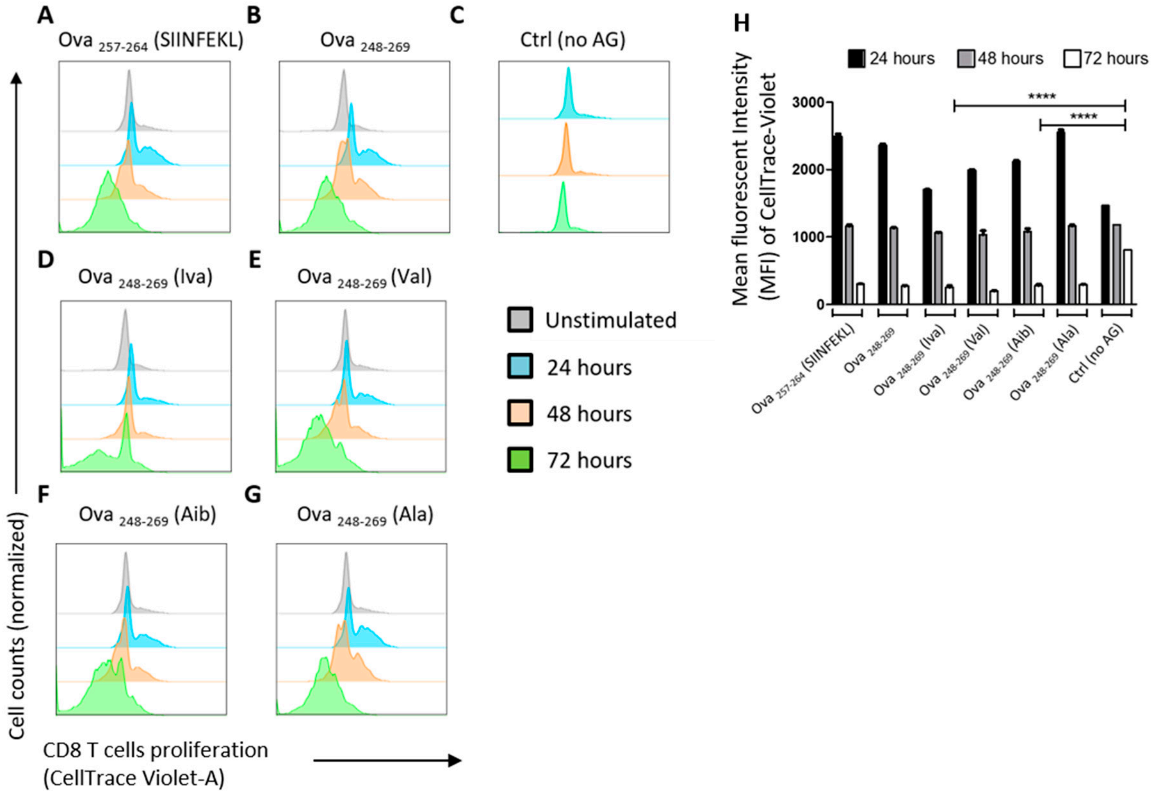


**Supplementary Figure 5: CD8 T cell proliferation dynamics.** CellTrace Violet labelled and LPS activated OT-1 mouse splenocytes stimulated with the tested peptides: the native ova-control peptides (A: short Ova $_{257\text{-}264}$ (SIINFEKL) or B: longer Ova $_{248\text{-}269}$), unstimulated (C) or with the ova peptides substituted with exo-amino acids (D: Ova $_{248\text{-}269}$ (Iva) and F: Ova $_{248\text{-}269}$ (Aib)) or peptides substituted natural amino acids (E: Ova $_{248\text{-}269}$ (Val) and G: Ova $_{248\text{-}269}$ (Ala). Cell proliferation was assessed via Flow cytometry by measuring reduction of the cell proliferation staining amongst CD8 T cell population after 24-, 48-, or 72 hours. A-G: Representative images show a histogram shift over time (blue: 24h, orange: 48h, green: 72h) that represent the reduction of the cell proliferation (CellTrace Violet) dye intensity amongst CD8 T cell population against the unstimulated control cells (grey histogram). H: Geometric Mean (CellTrace) of CD8 T cells. Three technical replicas were performed for each of the three biological replicas.

**SUPPORTING INFO**

## Contents


Peptide Synthesis protocols .......... 7

Ova$_{257-264}$ ; Native-short - H-SIINFEKL-OH .......... 7

Ova$_{248-269}$ ; Native-long - H-EVSGLEQLE-SIINFEKL-TEWTS-OH .......... 7

Ova$_{248-269}$ (Iva); Isovaline - H-EXSGXEQLX-SIINFEKL-XEXTS-OH .......... 8

Ova $_{248-269}$ (Val); Valine, Iva ctrl - H-EVSGVEQLV-SIINFEKL-VEVTS-OH .......... 9

Ova $_{248-269}$ (Aib); α-Aminoisobutyric acid - H-EYSGYEQLY-SIINFEKL-YEYTS-OH .......... 10

Ova $_{248-269}$ (Ala); Alanine, Aib ctrl - H-EASGAEQLA-SIINFEKL-AEATS-OH .......... 11

CEM liberty Blue synthesis cycle .......... 13

0.10-Resin Swelling (HS) .......... 13

0.10-Final Deprotection (HS) (UV) .......... 13

0.10-Single Coupling (HS) (UV) .......... 13

0.10-Double Coupling (HS) (UV) .......... 14

0.10-Single Coupling 4 min (HS) (UV) .......... 14

Preparative RP-HPLC methods and traces .......... 16

Ova$_{257-264}$ ; Native-short - H-SIINFEKL-OH .......... 16

Ova$_{248-269}$ ; Native-long - H-EVSGLEQLE-SIINFEKL-TEWTS-OH .......... 16

Ova$_{248-269}$ (Iva); Isovaline - H-EXSGXEQLX-SIINFEKL-XEXTS-OH .......... 17

Ova $_{248-269}$ (Val); Valine, Iva ctrl - H-EVSGVEQLV-SIINFEKL-VEVTS-OH .......... 17

Ova $_{248-269}$ (Aib); α-Aminoisobutyric acid - H-EYSGYEQLY-SIINFEKL-YEYTS-OH .......... 18

Ova $_{248-269}$ (Ala); Alanine, Aib ctrl - H-EASGAEQLA-SIINFEKL-AEATS-OH .......... 18

Analytical RP-HPLC-MS traces .......... 19

Ova$_{257-264}$ ; Native-short - H-SIINFEKL-OH .......... 19

Ova$_{248-269}$ ; Native-long - H-EVSGLEQLE-SIINFEKL-TEWTS-OH .......... 20

Ova$_{248-269}$ (Iva); Isovaline - H-EXSGXEQLX-SIINFEKL-XEXTS-OH .......... 21

Ova $_{248-269}$ (Val); Valine, Iva ctrl - H-EVSGVEQLV-SIINFEKL-VEVTS-OH .......... 22

Ova $_{248-269}$ (Aib); α-Aminoisobutyric acid - H-EYSGYEQLY-SIINFEKL-YEYTS-OH .......... 23

Ova $_{248-269}$ (Ala); Alanine, Aib ctrl - H-EASGAEQLA-SIINFEKL-AEATS-OH .......... 24

# Peptide Synthesis protocols

**Ova$_{257\text{-}264}$ ; Native-short -** H-**SIINFEKL**-OH

| Amino acid | Specific Derivative | Cycle |
|---|---|---|
| | Final Deprotection | 0.10-Final Deprotection (HS) (UV) |
| Ser - S | Fmoc-Ser(tBu)-OH | 0.10-Single Coupling (HS) (UV) |
| Ile - I | Fmoc-Ile-OH | 0.10-Single Coupling (HS) (UV) |
| Ile - I | Fmoc-Ile-OH | 0.10-Single Coupling (HS) (UV) |
| Asn - N | Fmoc-Asn(Trt)-OH | 0.10-Single Coupling (HS) (UV) |
| Phe - F | Fmoc-Phe-OH | 0.10-Single Coupling (HS) (UV) |
| Glu - E | Fmoc-Glu(OtBu)-OH | 0.10-Single Coupling (HS) (UV) |
| Lys - K | Fmoc-Lys(Boc)-OH | 0.10-Single Coupling (HS) (UV) |
| Leu - L | Fmoc-Leu-OH | 0.10-Resin Swelling (HS) |

**Ova$_{248\text{-}269}$ ; Native-long -** H-EVSGLEQLE-**SIINFEKL**-TEWTS-OH

| Amino acid | Specific Derivative | Cycle |
|---|---|---|
| | Final Deprotection | 0.10-Final Deprotection (HS) (UV) |
| Glu - E | Fmoc-Glu(OtBu)-OH | 0.10-Double Coupling (HS) (UV) |
| Val - V | Fmoc-Val-OH | 0.10-Double Coupling (HS) (UV) |
| Ser - S | Fmoc-Ser(tBu)-OH | 0.10-Single Coupling (HS) (UV) |
| Gly - G | Fmoc-Gly-OH | 0.10-Single Coupling (HS) (UV) |
| Leu - L | Fmoc-Leu-OH | 0.10-Single Coupling (HS) (UV) |
| Glu - E | Fmoc-Glu(OtBu)-OH | 0.10-Single Coupling (HS) (UV) |
| Gln - Q | Fmoc-Gln(Trt)-OH | 0.10-Single Coupling (HS) (UV) |
| Leu - L | Fmoc-Leu-OH | 0.10-Single Coupling (HS) (UV) |
| Glu - E | Fmoc-Glu(OtBu)-OH | 0.10-Single Coupling (HS) (UV) |
| Ser - S | Fmoc-Ser(tBu)-OH | 0.10-Single Coupling (HS) (UV) |
| Ile - I | Fmoc-Ile-OH | 0.10-Single Coupling (HS) (UV) |

| | | |
|---|---|---|
| Ile - I | Fmoc-Ile-OH | 0.10-Single Coupling (HS) (UV) |
| Asn - N | Fmoc-Asn(Trt)-OH | 0.10-Single Coupling (HS) (UV) |
| Phe - F | Fmoc-Phe-OH | 0.10-Single Coupling (HS) (UV) |
| Glu - E | Fmoc-Glu(OtBu)-OH | 0.10-Single Coupling (HS) (UV) |
| Lys - K | Fmoc-Lys(Boc)-OH | 0.10-Single Coupling (HS) (UV) |
| Leu - L | Fmoc-Leu-OH | 0.10-Single Coupling (HS) (UV) |
| Thr - T | Fmoc-Thr(tBu)-OH | 0.10-Single Coupling (HS) (UV) |
| Glu - E | Fmoc-Glu(OtBu)-OH | 0.10-Single Coupling (HS) (UV) |
| Trp - W | Fmoc-Trp(Boc)-OH | 0.10-Single Coupling (HS) (UV) |
| Thr - T | Fmoc-Thr(tBu)-OH | 0.10-Single Coupling (HS) (UV) |
| Ser - S | Fmoc-Ser(tBu)-Wang | 0.10-Resin Swelling (HS) |

**Ova$_{248\text{-}269}$ (Iva); Isovaline -** H-E**X**SG**X**EQL**X**-**SIINFEKL**-**X**E**X**TS-OH

| Amino acid | Specific Derivative | Cycle |
|---|---|---|
| | Final Deprotection | 0.10-Final Deprotection (HS) (UV) |
| Glu - E | Fmoc-Glu(OtBu)-OH | 0.10-Double Coupling (HS) (UV) |
| EX2 – X | Fmoc-Isovaline-OH | 0.10-Double Coupling (HS) (UV) |
| Ser - S | Fmoc-Ser(tBu)-OH | 0.10-Double Coupling (HS) (UV) |
| Gly - G | Fmoc-Gly-OH | 0.10-Double Coupling (HS) (UV) |
| EX2 - X | Fmoc-Isovaline-OH | 0.10-Single Coupling (HS) (UV) |
| Glu - E | Fmoc-Glu(OtBu)-OH | 0.10-Single Coupling (HS) (UV) |
| Gln - Q | Fmoc-Gln(Trt)-OH | 0.10-Double Coupling (HS) (UV) |
| Leu - L | Fmoc-Leu-OH | 0.10-Double Coupling (HS) (UV) |
| EX2 - X | Fmoc-Isovaline-OH | 0.10-Single Coupling (HS) (UV) |
| Ser - S | Fmoc-Ser(tBu)-OH | 0.10-Single Coupling (HS) (UV) |
| Ile - I | Fmoc-Ile-OH | 0.10-Single Coupling (HS) (UV) |
| Ile - I | Fmoc-Ile-OH | 0.10-Single Coupling (HS) (UV) |

| | | |
|---|---|---|
| Asn - N | Fmoc-Asn(Trt)-OH | 0.10-Single Coupling (HS) (UV) |
| Phe - F | Fmoc-Phe-OH | 0.10-Single Coupling (HS) (UV) |
| Glu - E | Fmoc-Glu(OtBu)-OH | 0.10-Single Coupling (HS) (UV) |
| Lys - K | Fmoc-Lys(Boc)-OH | 0.10-Double Coupling (HS) (UV) |
| Leu - L | Fmoc-Leu-OH | 0.10-Double Coupling (HS) (UV) |
| EX2 - X | Fmoc-Isovaline-OH | 0.10-Double Coupling (HS) (UV) |
| Glu - E | Fmoc-Glu(OtBu)-OH | 0.10-Double Coupling (HS) (UV) |
| EX2 - X | Fmoc-Isovaline-OH | 0.10-Single Coupling (HS) (UV) |
| Thr - T | Fmoc-Thr(tBu)-OH | 0.10-Single Coupling (HS) (UV) |
| Ser - S | Fmoc-Ser(tBu)-Wang | 0.10-Resin Swelling (HS) |

**Ova $_{248\text{-}269}$ (Val); Valine, Iva ctrl** - H-EVSGVEQLV-**SIINFEKL**-VEVTS-OH

| Amino acid | Specific Derivative | Cycle |
|---|---|---|
| | Final Deprotection | 0.10-Final Deprotection (HS) (UV) |
| Glu - E | Fmoc-Glu(OtBu)-OH | 0.10-Double Coupling (HS) (UV) |
| Val - V | Fmoc-Val-OH | 0.10-Double Coupling (HS) (UV) |
| Ser - S | Fmoc-Ser(tBu)-OH | 0.10-Single Coupling (HS) (UV) |
| Gly - G | Fmoc-Gly-OH | 0.10-Single Coupling (HS) (UV) |
| Val - V | Fmoc-Val-OH | 0.10-Single Coupling (HS) (UV) |
| Glu - E | Fmoc-Glu(OtBu)-OH | 0.10-Single Coupling (HS) (UV) |
| Gln - Q | Fmoc-Gln(Trt)-OH | 0.10-Single Coupling (HS) (UV) |
| Leu - L | Fmoc-Leu-OH | 0.10-Single Coupling (HS) (UV) |
| Val - V | Fmoc-Val-OH | 0.10-Single Coupling (HS) (UV) |
| Ser - S | Fmoc-Ser(tBu)-OH | 0.10-Single Coupling (HS) (UV) |
| Ile - I | Fmoc-Ile-OH | 0.10-Single Coupling (HS) (UV) |
| Ile - I | Fmoc-Ile-OH | 0.10-Single Coupling (HS) (UV) |
| Asn - N | Fmoc-Asn(Trt)-OH | 0.10-Single Coupling (HS) (UV) |

| | | |
|---|---|---|
| Phe - F | Fmoc-Phe-OH | 0.10-Single Coupling (HS) (UV) |
| Glu - E | Fmoc-Glu(OtBu)-OH | 0.10-Single Coupling (HS) (UV) |
| Lys - K | Fmoc-Lys(Boc)-OH | 0.10-Single Coupling (HS) (UV) |
| Leu - L | Fmoc-Leu-OH | 0.10-Single Coupling (HS) (UV) |
| Val - V | Fmoc-Val-OH | 0.10-Single Coupling (HS) (UV) |
| Glu - E | Fmoc-Glu(OtBu)-OH | 0.10-Single Coupling (HS) (UV) |
| Val - V | Fmoc-Val-OH | 0.10-Single Coupling (HS) (UV) |
| Thr - T | Fmoc-Thr(tBu)-OH | 0.10-Single Coupling (HS) (UV) |
| Ser - S | Fmoc-Ser(tBu)-Wang | 0.10-Resin Swelling (HS) |

**Ova $_{248\text{-}269}$ (Aib); α-Aminoisobutyric acid** - H-EYSGYEQLY**-SIINFEKL-**YEYTS-OH

| Amino acid | Specific Derivative | Cycle |
|---|---|---|
| | Final Deprotection | 0.10-Final Deprotection (HS) (UV) |
| Glu - E | Fmoc-Glu(OtBu)-OH | 0.10-Double Coupling (HS) (UV) |
| EX1 - B | Fmoc-Aib-OH | 0.10-Double Coupling (HS) (UV) |
| Ser - S | Fmoc-Ser(tBu)-OH | 0.10-Double Coupling (HS) (UV) |
| Gly - G | Fmoc-Gly-OH | 0.10-Double Coupling (HS) (UV) |
| EX1 - B | Fmoc-Aib-OH | 0.10-Single Coupling 4 min (HS) (UV) |
| Glu - E | Fmoc-Glu(OtBu)-OH | 0.10-Single Coupling (HS) (UV) |
| Gln - Q | Fmoc-Gln(Trt)-OH | 0.10-Double Coupling (HS) (UV) |
| Leu - L | Fmoc-Leu-OH | 0.10-Double Coupling (HS) (UV) |
| EX1 - B | Fmoc-Aib-OH | 0.10-Single Coupling 4 min (HS) (UV) |
| Ser - S | Fmoc-Ser(tBu)-OH | 0.10-Single Coupling (HS) (UV) |
| Ile - I | Fmoc-Ile-OH | 0.10-Single Coupling (HS) (UV) |
| Ile - I | Fmoc-Ile-OH | 0.10-Single Coupling (HS) (UV) |
| Asn - N | Fmoc-Asn(Trt)-OH | 0.10-Single Coupling (HS) (UV) |
| Phe - F | Fmoc-Phe-OH | 0.10-Single Coupling (HS) (UV) |
| Glu - E | Fmoc-Glu(OtBu)-OH | 0.10-Single Coupling (HS) (UV) |

| | | |
|---|---|---|
| Lys - K | Fmoc-Lys(Boc)-OH | 0.10-Double Coupling (HS) (UV) |
| Leu - L | Fmoc-Leu-OH | 0.10-Double Coupling (HS) (UV) |
| EX1 - B | Fmoc-Aib-OH | 0.10-Double Coupling (HS) (UV) |
| Glu - E | Fmoc-Glu(OtBu)-OH | 0.10-Double Coupling (HS) (UV) |
| EX1 - B | Fmoc-Aib-OH | 0.10-Single Coupling 4 min (HS) (UV) |
| Thr - T | Fmoc-Thr(tBu)-OH | 0.10-Single Coupling (HS) (UV) |
| Ser - S | Fmoc-Ser(tBu)-Wang | 0.10-Resin Swelling (HS) |

**Ova $_{248\text{-}269}$ (Ala); Alanine, Aib ctrl -** H-EASGAEQLA-**SIINFEKL-**AEATS-OH

| Amino acid | Specific Derivative | Cycle |
|---|---|---|
| | Final Deprotection | 0.10-Final Deprotection (HS) (UV) |
| Glu - E | Fmoc-Glu(OtBu)-OH | 0.10-Double Coupling (HS) (UV) |
| Ala - A | Fmoc-Ala-OH | 0.10-Double Coupling (HS) (UV) |
| Ser - S | Fmoc-Ser(tBu)-OH | 0.10-Single Coupling (HS) (UV) |
| Gly - G | Fmoc-Gly-OH | 0.10-Single Coupling (HS) (UV) |
| Ala - A | Fmoc-Ala-OH | 0.10-Single Coupling (HS) (UV) |
| Glu - E | Fmoc-Glu(OtBu)-OH | 0.10-Single Coupling (HS) (UV) |
| Gln - Q | Fmoc-Gln(Trt)-OH | 0.10-Single Coupling (HS) (UV) |
| Leu - L | Fmoc-Leu-OH | 0.10-Single Coupling (HS) (UV) |
| Ala - A | Fmoc-Ala-OH | 0.10-Single Coupling (HS) (UV) |
| Ser - S | Fmoc-Ser(tBu)-OH | 0.10-Single Coupling (HS) (UV) |
| Ile - I | Fmoc-Ile-OH | 0.10-Single Coupling (HS) (UV) |
| Ile - I | Fmoc-Ile-OH | 0.10-Single Coupling (HS) (UV) |
| Asn - N | Fmoc-Asn(Trt)-OH | 0.10-Single Coupling (HS) (UV) |
| Phe - F | Fmoc-Phe-OH | 0.10-Single Coupling (HS) (UV) |
| Glu - E | Fmoc-Glu(OtBu)-OH | 0.10-Single Coupling (HS) (UV) |

| | | |
|---|---|---|
| Lys - K | Fmoc-Lys(Boc)-OH | 0.10-Single Coupling (HS) (UV) |
| Leu - L | Fmoc-Leu-OH | 0.10-Single Coupling (HS) (UV) |
| Ala - A | Fmoc-Ala-OH | 0.10-Single Coupling (HS) (UV) |
| Glu - E | Fmoc-Glu(OtBu)-OH | 0.10-Single Coupling (HS) (UV) |
| Ala - A | Fmoc-Ala-OH | 0.10-Single Coupling (HS) (UV) |
| Thr - T | Fmoc-Thr(tBu)-OH | 0.10-Single Coupling (HS) (UV) |
| Ser - S | Fmoc-Ser(tBu)-Wang | 0.10-Resin Swelling (HS) |

# CEM liberty Blue synthesis cycle

## 0.10-Resin Swelling (HS)

| Step | Operation | Parameters | Pause |
|---|---|---|---|
| 1 | Swell Resin | Main solvent volume: 15<br>Time: 300 | False |

## 0.10-Final Deprotection (HS) (UV)

| Step | Operation | Parameters | Pause |
|---|---|---|---|
| 1 | Deprotection | ID: Standard Deprotection<br>Deprotection volume: 4.5 | False |
| 2 | Wash | Main solvent volume: 4<br>Time: 5.0<br>Drain type: UVDrain | False |
| 3 | Wash | Main solvent volume: 4<br>Time: 5.0<br>Drain type: UVDrain | False |
| 4 | Wash | Main solvent volume: 4<br>Time: 5.0<br>Drain type: Default | False |
| 5 | Wash | Main solvent volume: 4<br>Time: 5.0<br>Drain type: Default | False |

## 0.10-Single Coupling (HS) (UV)

| Step | Operation | Parameters | Pause |
|---|---|---|---|
| 1 | Deprotection | ID: Standard Deprotection<br>Deprotection volume: 4.5 | False |
| 2 | Wash | Main solvent volume: 4<br>Time: 15<br>Drain type: Default | False |
| 3 | Wash | Main solvent volume: 4<br>Time: 5<br>Drain type: Default | False |
| 4 | Wash | Main solvent volume: 4<br>Time: 5<br>Drain type: UVDrain | False |
| 5 | Wash | Main solvent volume: 4<br>Time: 3<br>Drain type: Default | False |
| 6 | Coupling | ID: Standard Coupling<br>Bottle position: (from method)<br>Bottle position volume: 2.5<br>Activator bottle position: PositionACT<br>Activator volume: 1<br>Activator base bottle position: PositionACTB<br>Activator base volume: 0.5<br>Delay reagent time: 0<br>Delay reagent bottle position: PositionACTB<br>Delay reagent volume: 0<br>Main solvent volume: 2<br>Value1: 1 | False |
| 7 | Wash Thru Manifold | Main solvent volume: 4<br>Time: 5<br>Drain type: Default | False |

| | | | |
|---|---|---|---|
| 8 | Wash | Main solvent volume: 4<br>Time: 5<br>Drain type: Default | False |

## 0.10-Double Coupling (HS) (UV)

| Step | Operation | Parameters | Pause |
|---|---|---|---|
| 1 | Deprotection | ID: Long Standard Deprotection<br>Deprotection volume: 4.5 | False |
| 2 | Wash | Main solvent volume: 4<br>Time: 15<br>Drain type: Default | False |
| 3 | Wash | Main solvent volume: 4<br>Time: 5<br>Drain type: Default | False |
| 4 | Wash | Main solvent volume: 4<br>Time: 5<br>Drain type: UVDrain | False |
| 5 | Wash | Main solvent volume: 4<br>Time: 5.0<br>Drain type: Default | False |
| 6 | Coupling | ID: Long Standard Coupling<br>Bottle position: (from method)<br>Bottle position volume: 2.5<br>Activator bottle position: PositionACT<br>Activator volume: 1<br>Activator base bottle position: PositionACTB<br>Activator base volume: 0.5<br>Delay reagent time: 0<br>Delay reagent bottle position: PositionACTB<br>Delay reagent volume: 0<br>Main solvent volume: 2<br>Value1: 1 | False |
| 7 | Coupling | ID: Long Standard Coupling<br>Bottle position: (from method)<br>Bottle position volume: 2.5<br>Activator bottle position: PositionACT<br>Activator volume: 1<br>Activator base bottle position: PositionACTB<br>Activator base volume: 0.5<br>Delay reagent time: 0<br>Delay reagent bottle position: PositionACTB<br>Delay reagent volume: 0<br>Main solvent volume: 2<br>Value1: 1 | False |
| 8 | Wash Thru Manifold | Main solvent volume: 4<br>Time: 5.0<br>Drain type: Default | False |
| 9 | Wash | Main solvent volume: 4<br>Time: 5.0<br>Drain type: Default | False |

## 0.10-Single Coupling 4 min (HS) (UV)

| Step | Operation | Parameters | Pause |
|---|---|---|---|
| 1 | Deprotection | ID: Standard Deprotection<br>Deprotection volume: 4.5 | False |
| 2 | Wash | Main solvent volume: 4<br>Time: 15 | False |

| | | | |
|---|---|---|---|
| | | Drain type: Default | |
| 3 | Wash | Main solvent volume: 4<br>Time: 5<br>Drain type: Default | False |
| 4 | Wash | Main solvent volume: 4<br>Time: 5<br>Drain type: UVDrain | False |
| 5 | Wash | Main solvent volume: 4<br>Time: 3<br>Drain type: Default | False |
| 6 | Coupling | ID: Long Standard Coupling<br>Bottle position: (from method)<br>Bottle position volume: 2.5<br>Activator bottle position: PositionACT<br>Activator volume: 1<br>Activator base bottle position: PositionACTB<br>Activator base volume: 0.5<br>Delay reagent time: 0<br>Delay reagent bottle position: PositionACTB<br>Delay reagent volume: 0<br>Main solvent volume: 2<br>Value1: 1 | False |
| 7 | Wash Thru Manifold | Main solvent volume: 4<br>Time: 5<br>Drain type: Default | False |
| 8 | Wash | Main solvent volume: 4<br>Time: 5<br>Drain type: Default | False |

# Preparative RP-HPLC methods and traces

**Ova$_{257-264}$ ; Native-short** - H-**SIINFEKL**-OH

Column: Phenomenex Luna C18(2) preparative column (5 µm. 100 Å, 21mm x250 mm D x L)

| Time<br>min | A - Water+ 0.1 % TFA<br>% | B - ACN+ 0.1 % TFA<br>% | Flow<br>mL/min |
|---|---|---|---|
| 0 | 75 | 25 | 20 |
| 12 | 50 | 50 | 20 |
| 15 | 0 | 100 | 20 |
| 18 | 0 | 100 | 20 |
| 21 | 75 | 25 | 20 |

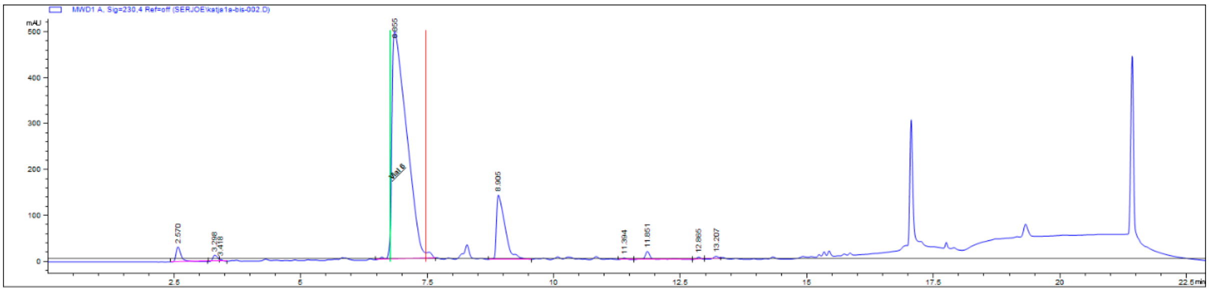


**R$_t$ =6.85 min**

**Ova$_{248-269}$ ; Native-long** - H-EVSGLEQLE-**SIINFEKL**-TEWTS-OH

Column: Phenomenex Jupiter C18 preparative column (5 µm. 300 Å, 21mm x250 mm D x L)

| Time<br>min | A - Water+ 0.1 % TFA<br>% | B - ACN+ 0.1 % TFA<br>% | Flow<br>mL/min |
|---|---|---|---|
| 0 | 75 | 25 | 20 |
| 15 | 50 | 50 | 20 |
| 16 | 0 | 100 | 20 |
| 19 | 0 | 100 | 20 |
| 21 | 75 | 25 | 20 |

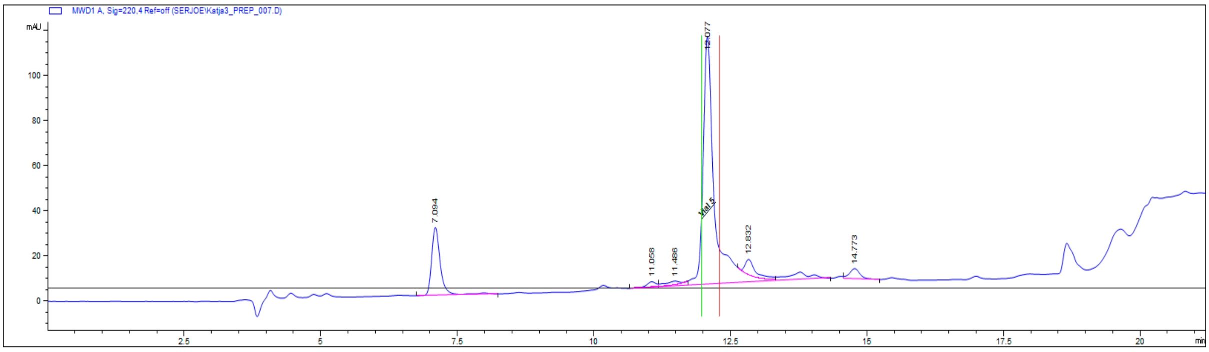


**R$_t$ = 12.08 min**

**Ova$_{248-269}$ (Iva); Isovaline** - H-E**X**SG**X**EQL**X-SIINFEKL-X**E**X**TS-OH

Column: Phenomenex Jupiter C18 preparative column (5 µm. 300 Å, 21mm x250 mm D x L)

| Time<br>min | A - Water+ 0.1 % TFA<br>% | B - ACN+ 0.1 % TFA<br>% | Flow<br>mL/min |
|---|---|---|---|
| 0 | 75 | 25 | 20 |
| 20 | 50 | 50 | 20 |
| 21 | 0 | 100 | 20 |
| 24 | 0 | 100 | 20 |
| 25 | 75 | 25 | 20 |

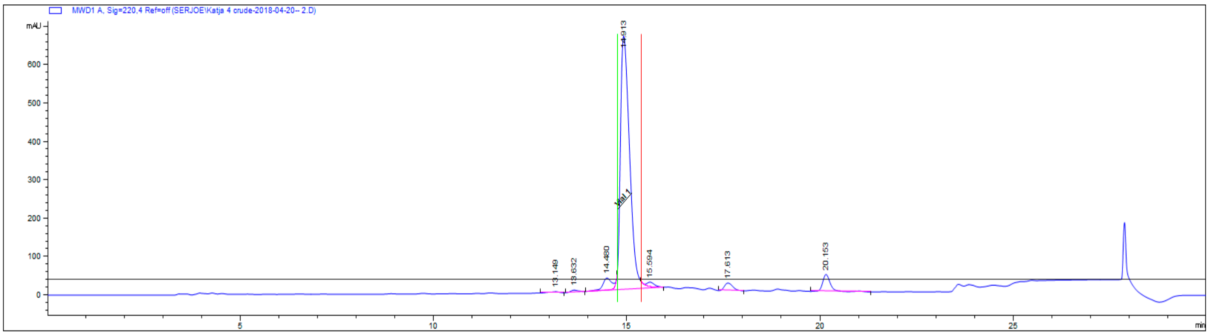


**R$_t$ = 14.9 min**

**Ova $_{248-269}$ (Val); Valine, Iva ctrl** - H-E**V**SG**V**EQL**V-SIINFEKL-V**E**V**TS-OH

Column: Phenomenex Jupiter C18 preparative column (5 µm. 300 Å, 21mm x250 mm D x L)

| Time<br>min | A - Water+ 0.1 % TFA<br>% | B - ACN+ 0.1 % TFA<br>% | Flow<br>mL/min |
|---|---|---|---|
| 0 | 75 | 25 | 20 |
| 15 | 50 | 50 | 20 |
| 16 | 0 | 100 | 20 |
| 19 | 0 | 100 | 20 |
| 21 | 75 | 25 | 20 |

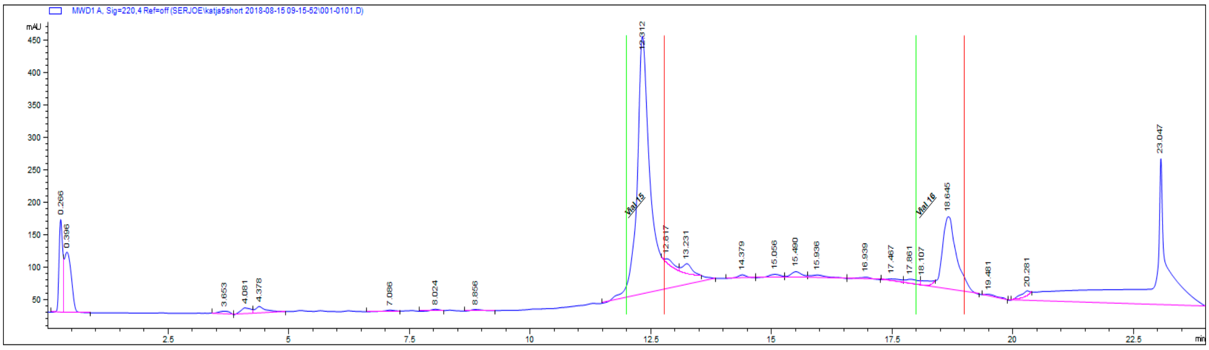


**R$_t$ = 12.31 min**

**Ova $_{248\text{-}269}$ (Aib); α-Aminoisobutyric acid** - H-EYSGYEQLY-**SIINFEKL**-YEYTS-OH

Column: Phenomenex Jupiter C18 preparative column (5 µm. 300 Å, 21mm x250 mm D x L)

| Time<br>min | A - Water+ 0.1 % TFA<br>% | B - ACN+ 0.1 % TFA<br>% | Flow<br>mL/min |
|---|---|---|---|
| 0 | 75 | 25 | 20 |
| 20 | 50 | 50 | 20 |
| 21 | 0 | 100 | 20 |
| 24 | 0 | 100 | 20 |
| 25 | 75 | 25 | 20 |

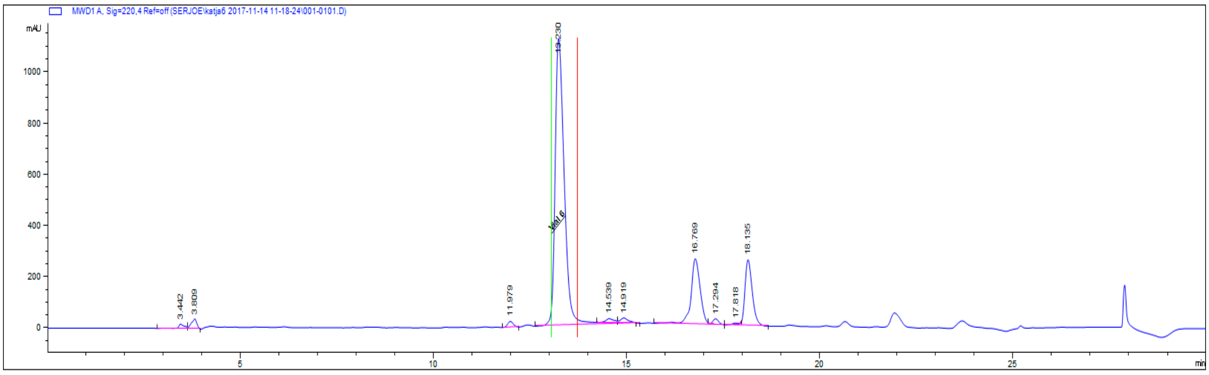


**$R_t$ = 13.23 min**

**Ova $_{248\text{-}269}$ (Ala); Alanine, Aib ctrl** - H-EASGAEQLA-**SIINFEKL**-AEATS-OH

Column: Phenomenex Jupiter C18 preparative column (5 µm. 300 Å, 21mm x250 mm D x L)

| Time<br>min | A - Water+ 0.1 % TFA<br>% | B - ACN+ 0.1 % TFA<br>% | Flow<br>mL/min |
|---|---|---|---|
| 0 | 75 | 25 | 20 |
| 15 | 50 | 50 | 20 |
| 16 | 0 | 100 | 20 |
| 19 | 0 | 100 | 20 |
| 21 | 75 | 25 | 20 |

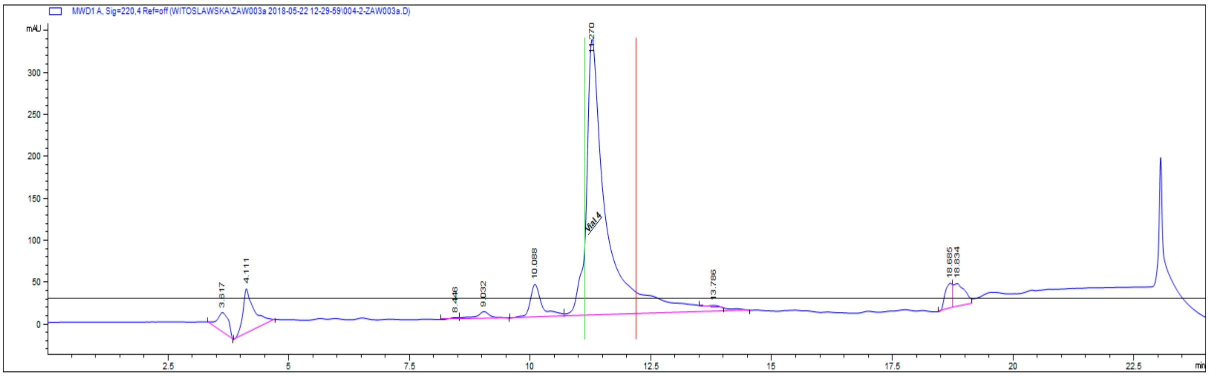


**$R_t$ = 11.27 min**

# Analytical RP-HPLC-MS traces

**Ova$_{257\text{-}264}$ ; Native-short** - H-**SIINFEKL**-OH

Column: Phenomenex Luna C18(2) analytical column (5 µm. 100 Å, 4.6mm x250 mm D x L)

| Time<br>min | A - Water+ 0.1 % TFA<br>% | B - ACN+ 0.1 % TFA<br>% | Flow<br>mL/min |
|---|---|---|---|
| 0 | 85 | 15 | 1 |
| 45 | 0 | 100 | 1 |
| 50 | 0 | 100 | 1 |
| 58 | 85 | 15 | 1 |

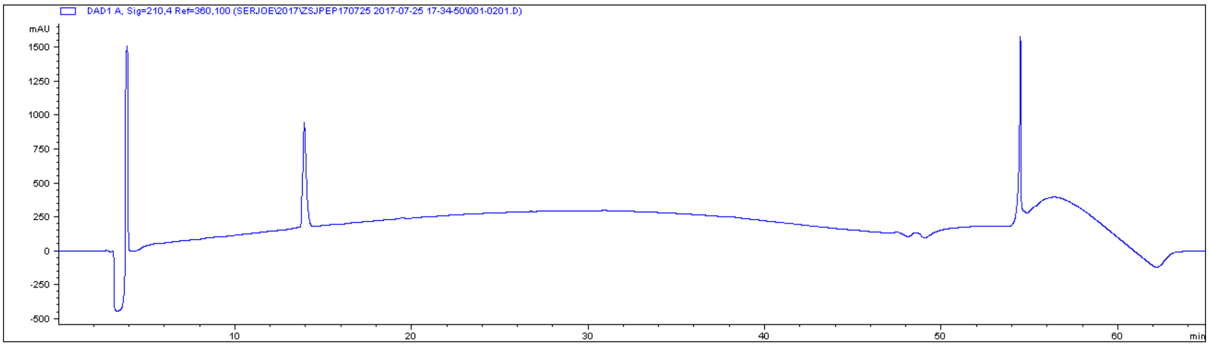


**UV 210 nm - R$_t$ =13.9 min - purity 99%**

MS spectrum of peak at 13.9 min

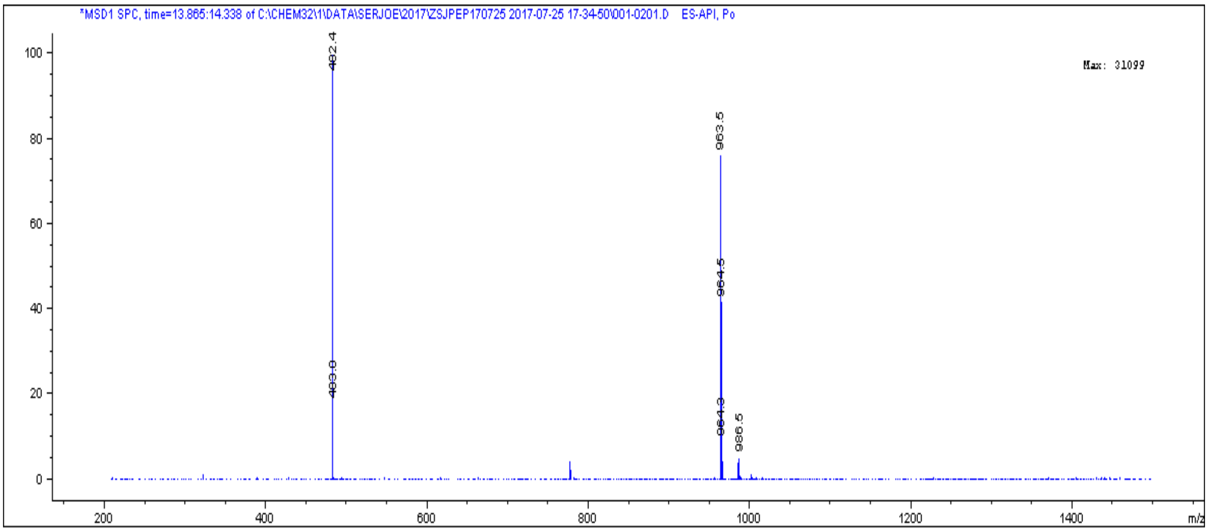


Mass calculated for $C_{45}H_{75}N_{10}O_{13}$ $[M+H]^{+}$ = 963.54, measured 963.5
Mass calculated for $C_{45}H_{76}N_{10}O_{13}/2$ $[M+2H]^{2+}/2$ = 965.56/2 = 482.78, measured 482.4

**Ova$_{248\text{-}269}$ ; Native-long** - H-EVSGLEQLE-**SIINFEKL**-TEWTS-OH

Column: Phenomenex Jupiter C18 analytical column (5 µm. 300 Å, 4.6mm x150 mm D x L)

| Time<br>min | A - Water+ 0.1 % TFA<br>% | B - ACN+ 0.1 % TFA<br>% | Flow<br>mL/min |
|---|---|---|---|
| 0 | 85 | 15 | 1 |
| 30 | 0 | 100 | 1 |
| 33 | 0 | 100 | 1 |
| 36 | 85 | 15 | 1 |

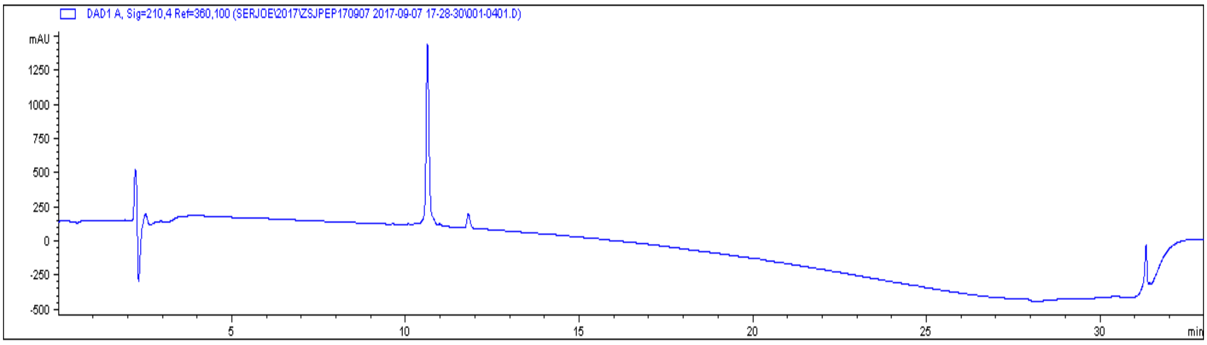


**UV 210 nm - R$_t$ =10.6 min - purity 93%**

MS spectrum of peak at 10.63 min

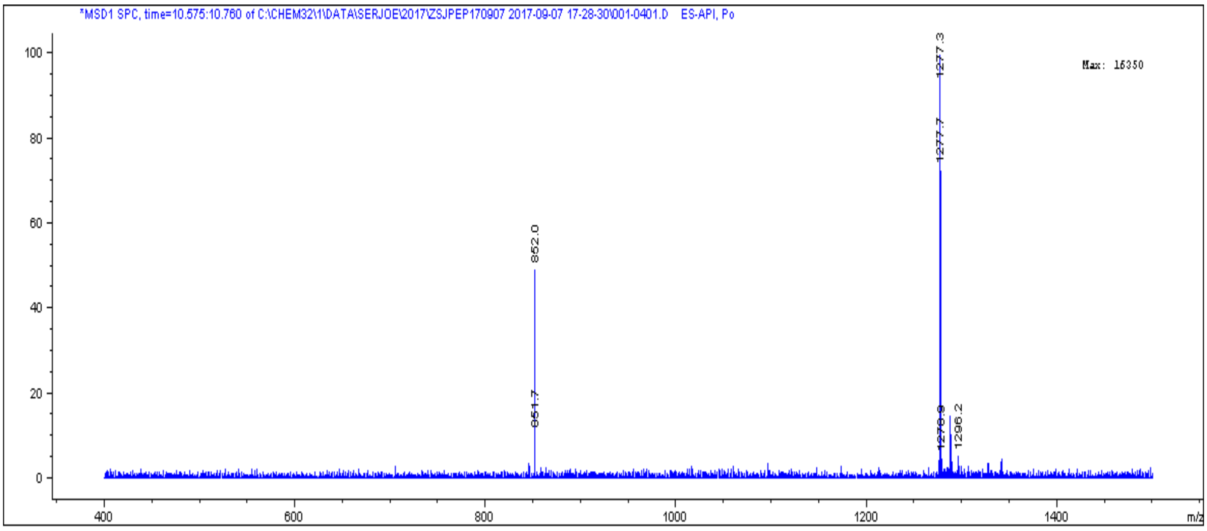


Molecular weight calculated for $C_{114}H_{179}N_{26}O_{40}$/2 $[M+2H]^{2+}/2$ = 2553.83/2 = 1276.9, measured 1277.3
Molecular weight calculated for $C_{114}H_{180}N_{26}O_{40}$/3 $[M+3H]^{3+}/3$ = 2554.83/3 = 851.61, measured 852.0
Molecular weight calculated for $C_{114}H_{179}N_{26}O_{40}$ = 2552.82, deconvoluted mass = 2552.78

**Ova$_{248-269}$ (Iva); Isovaline** - H-EXSGXEQLX-**SIINFEKL**-XEXTS-OH

Column: Phenomenex Jupiter C18 analytical column (5 µm. 300 Å, 4.6mm x150 mm D x L)

| Time<br>min | A - Water+ 0.1 % TFA<br>% | B - ACN+ 0.1 % TFA<br>% | Flow<br>mL/min |
|---|---|---|---|
| 0 | 85 | 15 | 1 |
| 30 | 0 | 100 | 1 |
| 33 | 0 | 100 | 1 |
| 36 | 85 | 15 | 1 |

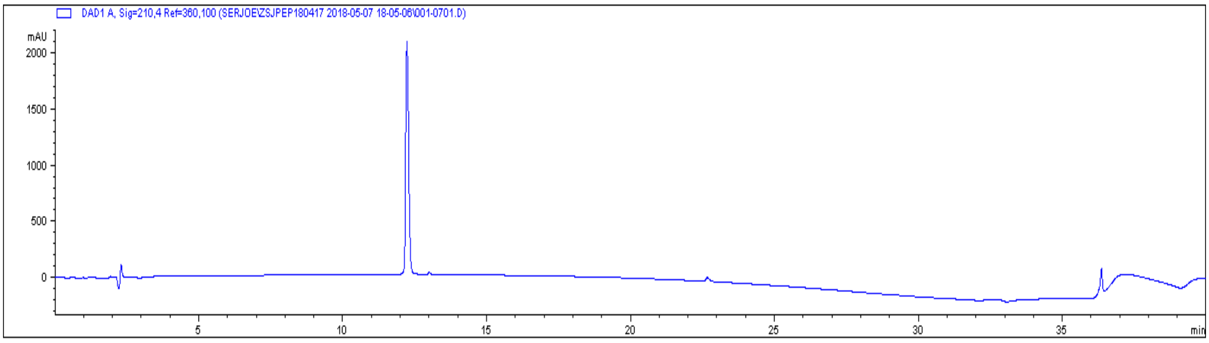


**UV 210 nm - R$_t$ =12.2 min - purity 99%**

MS spectrum of peak at 12.2 min

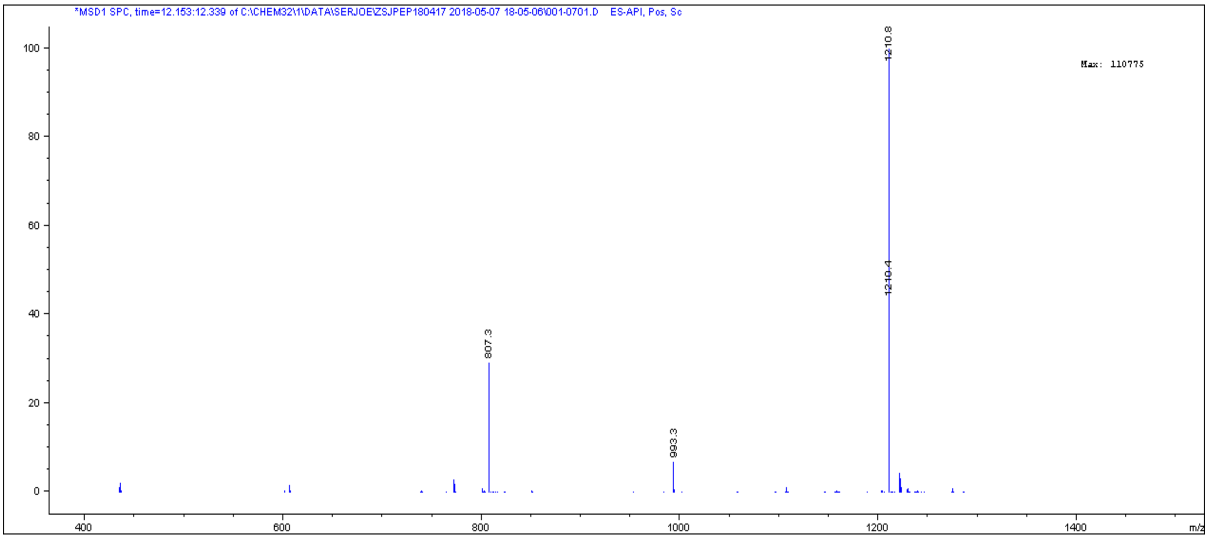


Molecular weight calculated for $C_{114}H_{179}N_{26}O_{40}$/2 $[M+2H]^{2+}$/2 = 2420.77/2 = 1210.38, measured 1210.8
Molecular weight calculated for $C_{114}H_{180}N_{26}O_{40}$/3 $[M+3H]^{3+}$/3 = 2421.77/3 = 807.25, measured 807.3
Molecular weight calculated for $C_{114}H_{179}N_{26}O_{40}$ = 2419.76, deconvoluted mass = 2419.23

**Ova $_{248\text{-}269}$ (Val); Valine, Iva ctrl** - H-EVSGVEQLV-**SIINFEKL**-VEVTS-OH

Column: Phenomenex Jupiter C18 analytical column (5 µm. 300 Å, 4.6mm x150 mm D x L)

| Time<br>min | A - Water+ 0.1 % TFA<br>% | B - ACN+ 0.1 % TFA<br>% | Flow<br>mL/min |
|---|---|---|---|
| 0 | 95 | 5 | 1 |
| 30 | 0 | 100 | 1 |
| 33 | 0 | 100 | 1 |
| 36 | 95 | 5 | 1 |

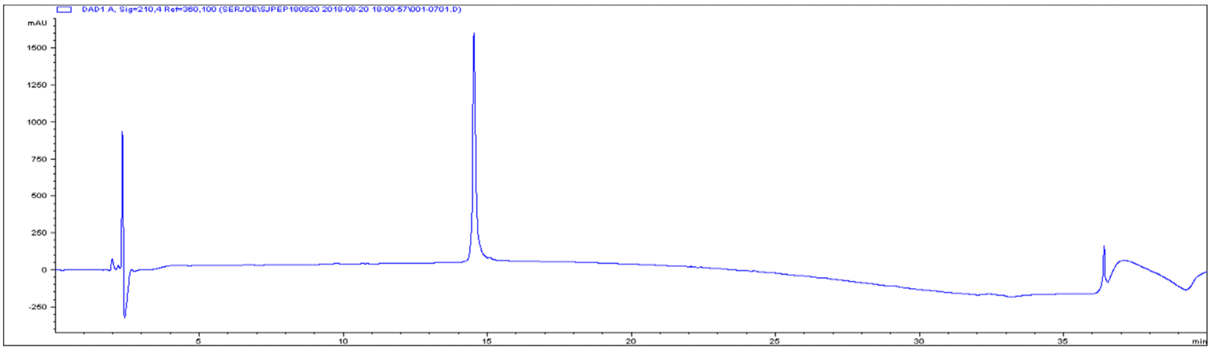


**UV 210 nm - $R_t$ =14.5 min - purity 98%**

MS spectrum of peak at 14.5 min

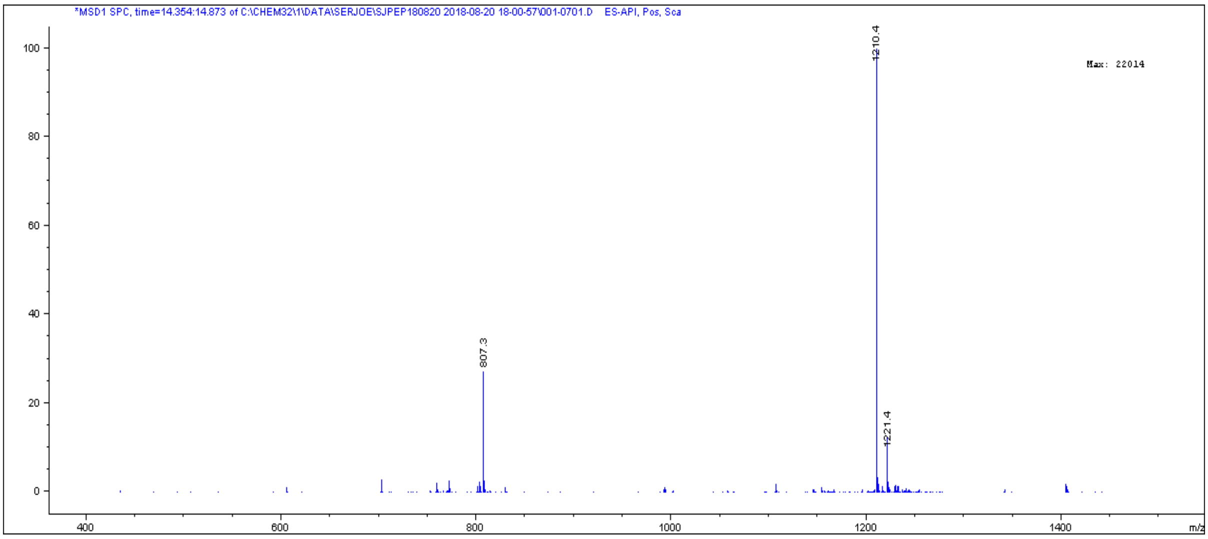


Molecular weight calculated for $C_{114}H_{179}N_{26}O_{40}/2$ $[M+2H]^{2+}/2$ = 2420.77/2 = 1210.38, measured 1210.8
Molecular weight calculated for $C_{114}H_{180}N_{26}O_{40}/3$ $[M+3H]^{3+}/3$ = 2421.77/3 = 807.25, measured 807.3
Molecular weight calculated for $C_{114}H_{179}N_{26}O_{40}$ = 2419.76, deconvoluted mass = 2419.23

**Ova $_{248\text{-}269}$ (Aib); α-Aminoisobutyric acid** - H-EYSGYEQLY-**SIINFEKL**-YEYTS-OH

Column: Phenomenex Jupiter C18 analytical column (5 µm. 300 Å, 4.6mm x150 mm D x L)

| Time<br>min | A - Water+ 0.1 % TFA<br>% | B - ACN+ 0.1 % TFA<br>% | Flow<br>mL/min |
|---|---|---|---|
| 0 | 85 | 15 | 1 |
| 30 | 0 | 100 | 1 |
| 33 | 0 | 100 | 1 |
| 36 | 85 | 15 | 1 |

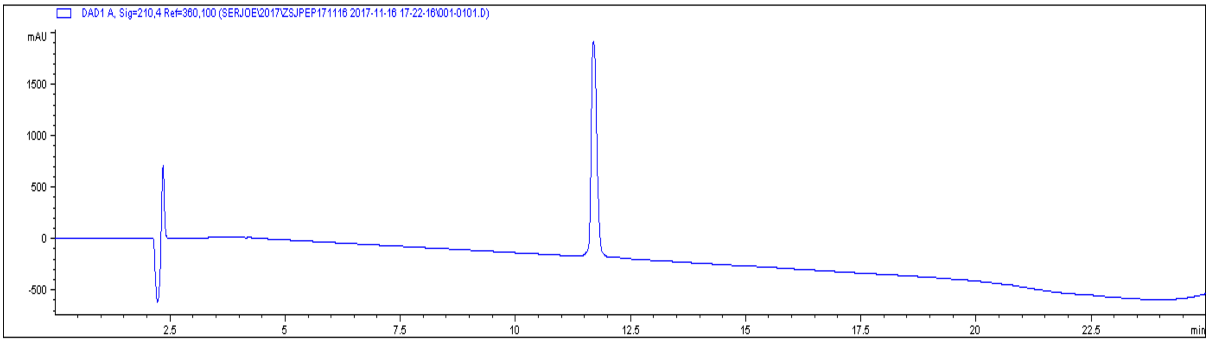


**UV 210 nm - $R_t$ =15.3 min - purity 99%**

MS spectrum of peak at 15.3 min

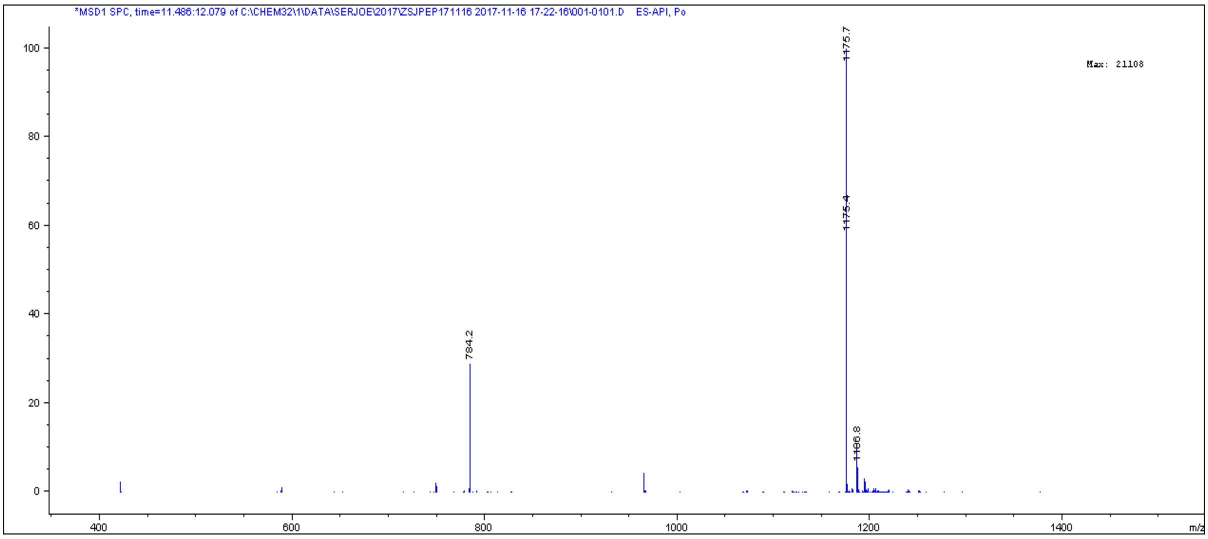


Molecular weight calculated for $C_{114}H_{179}N_{26}O_{40}$/2 $[M+2H]^{2+}$/2 = 2350.63/2 = 1175.32, measured 1175.7
Molecular weight calculated for $C_{114}H_{180}N_{26}O_{40}$/3 $[M+3H]^{3+}$/3 = 2351.64/3 = 783.88, measured 784.2
Molecular weight calculated for $C_{114}H_{179}N_{26}O_{40}$ = 2349.62, deconvoluted mass = 2349.48

**Ova 248-269 (Ala); Alanine, Aib ctrl** - H-EASGAEQLA-**SIINFEKL**-AEATS-OH

Column: Phenomenex Jupiter C18 analytical column (5 µm. 300 Å, 4.6mm x150 mm D x L)

| Time<br>min | A - Water+ 0.1 % TFA<br>% | B - ACN+ 0.1 % TFA<br>% | Flow<br>mL/min |
|---|---|---|---|
| 0 | 95 | 5 | 1 |
| 30 | 0 | 100 | 1 |
| 33 | 0 | 100 | 1 |
| 36 | 95 | 5 | 1 |

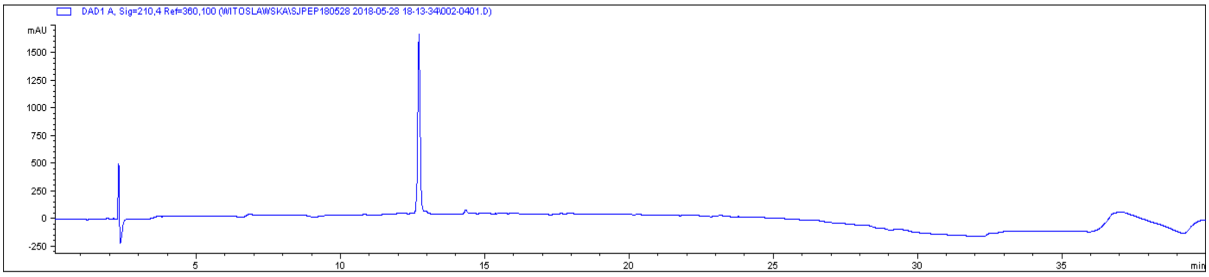


**UV 210 nm - $R_t$ =15.3 min - purity 98%**

MS spectrum of peak at 15.3 min

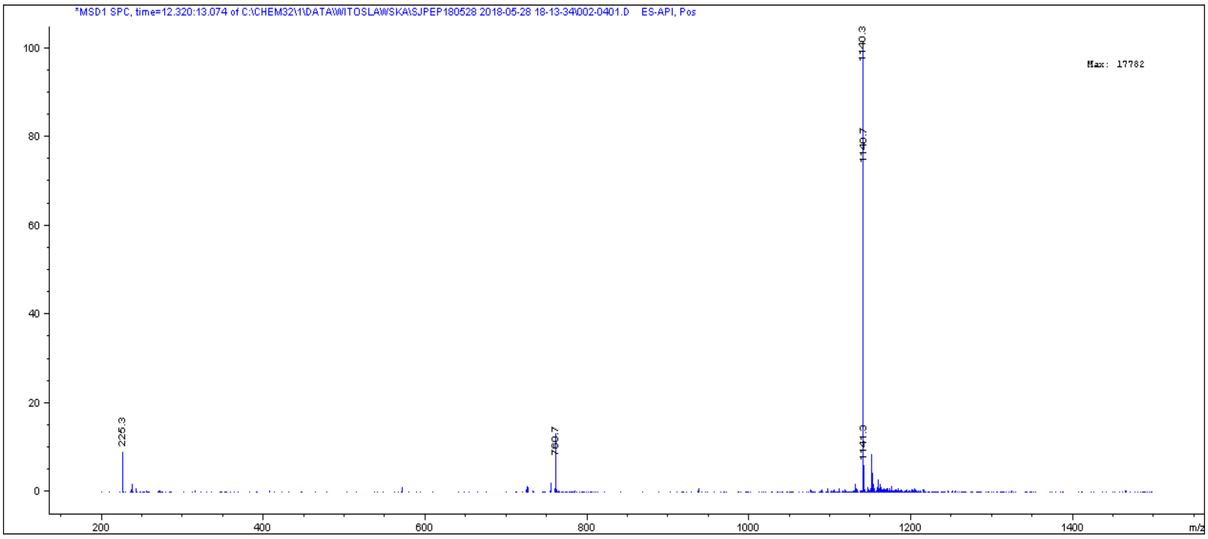


Molecular weight calculated for $C_{114}H_{179}N_{26}O_{40}$/2 $[M+2H]^{2+}$/2 = 2281.51/2 = 1140.76, measured 1140.3
Molecular weight calculated for $C_{114}H_{180}N_{26}O_{40}$/3 $[M+3H]^{3+}$/3 = 2282.52/3 = 760.84, measured 760.7
Molecular weight calculated for $C_{114}H_{179}N_{26}O_{40}$ = 2279.49, deconvoluted mass = 2278.83
